# Modeling and simulation of the redox regulation of the metabolism in *Escherichia coli* at different oxygen concentrations

**DOI:** 10.1186/s13068-017-0867-0

**Published:** 2017-07-14

**Authors:** Yu Matsuoka, Hiroyuki Kurata

**Affiliations:** 1 Department of Bioscience and Bioinformatics, Kyushu Institute of Technology, 680-4 Kawazu, Iizuka, Fukuoka, 820-8502 Japan; 2 Biomedical Informatics R&D Center, Kyushu Institute of Technology, 680-4 Kawazu, Iizuka, Fukuoka, 820-8502 Japan

**Keywords:** Kinetic modeling, Fermentation, Dissolved oxygen limitation, Redox regulation, ArcA, Fnr, Respiratory chain, NADH/NAD^+^ ratio, *Escherichia coli*

## Abstract

**Background:**

Microbial production of biofuels and biochemicals from renewable feedstocks has received considerable recent attention from environmental protection and energy production perspectives. Many biofuels and biochemicals are produced by fermentation under oxygen-limited conditions following initiation of aerobic cultivation to enhance the cell growth rate. Thus, it is of significant interest to investigate the effect of dissolved oxygen concentration on redox regulation in *Escherichia coli*, a particularly popular cellular factory due to its high growth rate and well-characterized physiology. For this, the systems biology approach such as modeling is powerful for the analysis of the metabolism and for the design of microbial cellular factories.

**Results:**

Here, we developed a kinetic model that describes the dynamics of fermentation by taking into account transcription factors such as ArcA/B and Fnr, respiratory chain reactions and fermentative pathways, and catabolite regulation. The hallmark of the kinetic model is its ability to predict the dynamics of metabolism at different dissolved oxygen levels and facilitate the rational design of cultivation methods. The kinetic model was verified based on the experimental data for a wild-type *E. coli* strain. The model reasonably predicted the metabolic characteristics and molecular mechanisms of *fnr* and *arcA* gene-knockout mutants. Moreover, an aerobic–microaerobic dual-phase cultivation method for lactate production in a *pfl*-knockout mutant exhibited promising yield and productivity.

**Conclusions:**

It is quite important to understand metabolic regulation mechanisms from both scientific and engineering points of view. In particular, redox regulation in response to oxygen limitation is critically important in the practical production of biofuel and biochemical compounds. The developed model can thus be used as a platform for designing microbial factories to produce a variety of biofuels and biochemicals.

**Electronic supplementary material:**

The online version of this article (doi:10.1186/s13068-017-0867-0) contains supplementary material, which is available to authorized users.

## Background

Microbial production of biofuels and biochemicals from renewable feedstocks has received considerable recent attention from environmental protection and energy production perspectives. A limited number of cell factory platforms have been employed for the industrial production of a wide range of fuels and chemicals. *Escherichia coli* is probably the most widely used cellular factory due to its high growth rate and well-characterized physiology [1]. Many biofuels and biochemicals, such as ethanol and lactate, are produced by fermentation under oxygen-limited conditions. One method in particular, dual-phase cultivation method, combines the advantages afforded by aerobic and micro-aerobic (or anaerobic) conditions [2, 3]. In dual-phase processes, cultivation is initiated with an aerobic culture to increase the biomass (contributing to productivity), and it is followed by anaerobic or micro-aerobic cultivation to facilitate efficient production of the target product. It is, therefore, highly desirable to evaluate the metabolic characteristics at different dissolved oxygen (DO) concentrations. For this purpose, appropriate quantitative models that can simulate such cultivations are needed.

Of the various modeling approaches currently available to cellular metabolism, flux balance analysis (FBA) approach has been extensively employed, but restricts to stoichiometric equations at the steady state, and thus it is difficult to simulate the dynamic changes in metabolic fluxes. On the other hand, a kinetic modeling approach can reproduce the dynamics of metabolite concentrations and fluxes in response to changes in genetic and environmental conditions [4], because it takes into account the mechanism of complex reactions such as allosteric modulation [5, 6], enzyme modification [7], and gene expression regulation by transcription factors (TFs) [8–11].

Of the various types of metabolic regulation, carbon catabolite regulation has been extensively modeled by a number of researchers [7, 12–19] to elucidate the mechanism of carbon uptake and metabolism. A detailed kinetic model of central carbon metabolism in *E. coli* that incorporates a constrained optimization method for parameter estimation on a supercomputer was recently developed [20]. As compared with other kinetic models, this model enabled more accurate prediction of the dynamics of wild-type (WT) cells and multiple-gene-knockout mutants in batch culture. However, from the perspective of practical applications to develop cellular factories for biofuel and biochemical production, the effect of oxygen limitation on redox regulation with carbon catabolite regulation is critically important. Considerable effort has been expended in this regard in the Systems Understanding of Microbial Oxygen Metabolism (SUMO) project [21–27].

For the proper modeling on the respiratory chain and the redox regulation, we have to consider the basic regulation mechanisms. Oxygen serves as the final electron acceptor of the respiratory chain [28]. In *E. coli*, two major oxidases, cytochrome *bo* (Cyo) and cytochrome *bd* (Cyd), transfer electrons from quinol to oxygen [29, 30]. Cyo has a low affinity for oxygen but a high reaction rate and functions primarily under aerobic conditions. By contrast, Cyd has high oxygen affinity but a lower reaction rate and functions primarily under micro-aerobic conditions. On the other hand, the dehydrogenases NADH dehydrogenase-I (Nuo) and NADH dehydrogenase-II (Ndh) oxidize electron donors such as NADH and FADH_2_ by reducing quinone to quinol [30]. The function of the respiratory chain is the successive transport of electrons from electron donors to electron acceptors with translocation of protons from the cytoplasm to the periplasmic space via the inner membrane. The resulting proton gradient (proton motive force) drives ATP synthesis. This series of reactions proceeds when oxygen is available, such as under aerobic or micro-aerobic conditions.

At limited oxygen concentrations, the transcription factors Fnr and ArcA/B play essential roles in metabolic regulation in *E. coli* [21]. The direct oxygen sensor Fnr regulates the expression of metabolic pathway genes under anaerobic conditions [31], whereas ArcA/B regulates these genes under both micro-aerobic and anaerobic conditions [32, 33]. The ArcA/B system is a two-component system: ArcB is a membrane-bound sensor kinase and ArcA is the cognate response regulator. ArcB auto-phosphorylates, and then *trans*-phosphorylates ArcA when oxygen is limited [34]. Phosphorylated ArcA in turn either activates or represses the expression of metabolic pathway genes. In addition, phosphorylated ArcA represses *cyoABCD*, which encodes Cyo, and activates *cydAB*, which encodes Cyd in the respiratory chain. Note that quinone inhibits the auto-phosphorylation of ArcB [35], which in turn represses the activity of ArcA.

The redox ratio (i.e., NADH/NAD^+^) increases as the activity of the respiratory chain decreases in response to oxygen limitation. The excretion rates of fermentation products such as lactate, ethanol, succinate, formate (CO_2_ and H_2_ also), and acetate are influenced by this redox ratio. NADH is reoxidized to generate NAD^+^ via these fermentative pathways to enable continuation of metabolism under micro-aerobic and anaerobic conditions. Lactate is formed by lactate dehydrogenase (LDH), whereas ethanol is formed by acetaldehyde dehydrogenase (ALDH) and alcohol dehydrogenase (ADH). Succinate is formed from phosphoenol pyruvate (PEP) via phosphoenolpyruvate carboxylase (Ppc) through the reverse pathway of the normal tricarboxylic acid (TCA) cycle from oxaloacetate to succinate, whereas the succinate dehydrogenase (SDH) pathway is reversed by fumarate reductase (Frd). Formate is formed by pyruvate formate-lyase (Pfl), and acetate is formed by phosphoacetyl transferase (Pta) and acetate kinase (Ack).

In the present study, we developed a kinetic model that describes the dynamics of the metabolism in response to different DO levels by taking into account the roles of transcription factors such as ArcA/B and Fnr, the respiratory chain reactions, the fermentative pathways as mentioned above, as well as catabolite regulation.

## Methods

### Modeling primary metabolism

**Fig. 1:**
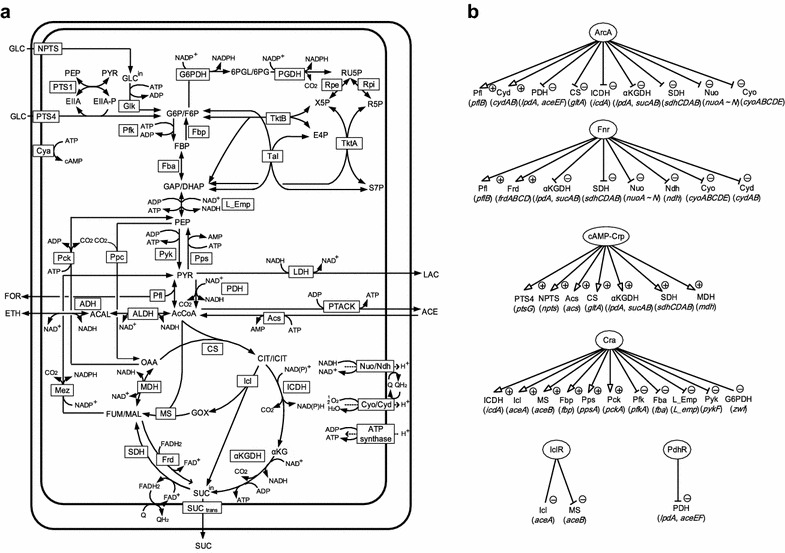
Metabolic network and transcriptional regulation in *Escherichia coli*. Primary metabolic pathways (**a**) and gene regulation (**b**)

Figure 1 shows the primary metabolic pathways of *E. coli*, including glycolysis, TCA cycle, pentose phosphate (PP), gluconeogenic, glyoxylate, and anaplerotic pathways, as well as the substrate transport system such as phosphotransferase system (PTS). Kinetic models of these pathways have been developed to investigate carbon uptake and metabolism under aerobic conditions [13–15, 17, 20]. Here, we constructed a kinetic model applicable under micro-aerobic (and anaerobic) conditions as well. For this purpose, the model incorporates additional fermentative pathways including such enzymes as LDH, Pfl, and ADH. We also considered respiratory chain mediators such as Nuo, Ndh, Cyo, and Cyd and redox regulation by Fnr and ArcA/B in response to changes in DO level. The detailed mass balance equations and the kinetic models are given in Additional file 1.

Once the overall metabolic fluxes of primary metabolism are calculated, the specific ATP, specific CO_2_, and specific NAD(P)H production rates can be estimated. ATP is produced via either substrate-level phosphorylation or oxidative phosphorylation. Referring to Fig. 1, the specific ATP production rate can be expressed as follows:

1 $$v_{{{\text{ATP}}}} = {\text{OP}} + v_{{{\text{L}}\_{\text{Emp}}}} + v_{{{\text{Pyk}}}} + v_{{{\text{PTACK}}}} + v_{{\alpha {\text{KGDH}}}} - v_{{{\text{Glk}}}} - v_{{{\text{Pfk}}}} - v_{{{\text{Pps}}}} - v_{{{\text{Pck}}}} - v_{{{\text{Acs}}}}.$$

Note that L_Emp is the lumped pathway from glyceraldehyde-3-phosphate/dihydroxy acetone phosphate (GAP/DHAP) to PEP, and PTACK is the combined pathway for Pta and Ack (Fig. 1). In Eq. 1, OP represents the specific ATP production rate via oxidative phosphorylation, which can be estimated by introducing the H^+^/ATP ratio, which indicates the ratio of proton transport-coupled ATP synthesis, where H^+^/ATP = 3 [36], and calculating the proton transfer rate via Nuo, Cyo, and Cyd reactions, such that

2 $${\text{OP}} = \frac{1}{3}\left( {4 \cdot v_{\text{Nuo}} + 4 \cdot v_{\text{Cyo}} + 2 \cdot v_{\text{Cyd}} } \right),$$

where the proton transfer efficiency, which is indicated as the number of protons delivered to the periplasmic side of the membrane per electron (H^+^/e^−^ ratio), is taken into account for each enzyme. The H^+^/e^−^ ratios for Nuo, Cyo, and Cyd can be estimated as 2, 2, and 1, respectively [28]. Note that NADH, which carries two electrons, is oxidized by dehydrogenases, and the electrons are subsequently transferred to cytochromes with subsequent conversion of oxygen to H_2_O.

The specific growth rate (*μ*) was estimated based on the experimental observation that cell growth and specific ATP production rates are linearly correlated [13, 37, 38]:

3 $$\mu = k_{\text{ATP}} \cdot v_{\text{ATP}},$$

where *k*_ATP_ represents the constant parameter, and *v*_ATP_ represents the specific ATP production rate computed using Eq. 1.

The specific CO_2_ production rate can be estimated by

4 $$\nu_{{{\text{CO}}_{ 2} }} = v_{\text{PGDH}} + v_{\text{PDH}} + v_{\text{ICDH}} + v_{{\alpha {\text{KGDH}}}} + v_{\text{Mez}} + v_{\text{Pck}} - v_{\text{Ppc}},$$

the specific NADPH production rate can be estimated by

5 $$\nu_{\text{NADPH}} = v_{\text{G6PDH}} + v_{\text{PGDH}} + v_{\text{ICDH}} + v_{\text{Mez}},$$

and the specific production/consumption rates of NADH can be estimated by

6 $$v_{{{\text{NADH}}}} = v_{{{\text{L}}\_{\text{Emp}}}} + v_{{{\text{PDH}}}} + v_{{\alpha {\text{KGDH}}}} + v_{{{\text{MDH}}}} - v_{{{\text{LDH}}}} - v_{{{\text{ALDH}}}} - v_{{{\text{ADH}}}} - v_{{{\text{Nuo}}}} - v_{{{\text{Ndh}}}}.$$

To properly model primary metabolism, the metabolic regulation mechanisms must be incorporated. Enzyme-level regulation can be represented by incorporating the effectors (metabolites) into the corresponding kinetic models. For example, in *E. coli*, fructose-1,6-bisphosphate (FBP) is the feed-forward activator of pyruvate kinase (Pyk) and Ppc, whereas PEP is the feedback inhibitor of phosphofructokinase (Pfk). These effectors were incorporated in the corresponding kinetic models (Additional file 1).

Transcriptional regulation is also important and can be represented by the TFs, such that

7 $$v_{ \cdot }^{\hbox{max} } = v_{ \cdot }^{{\max^{\prime } }} \cdot f\left( {{\text{TF}}_{i} } \right),$$

where TF_*i*_ represents the activity of the *i*th transcription factor, and *v*_∙_^max′^ represents the original maximum reaction rate for the corresponding pathway reaction. The detailed equations are given in Additional file 1.

In the redox regulation, Fnr and ArcA play important oxygen-dependent roles, with the activities of such TFs governed by cytoplasmic oxygen concentration [*O*_2_] and oxygenated quinone (after this, simply quinone) concentration [*Q*], respectively. The activities of Fnr and ArcA can be expressed as Hill equation [39] as follows:

8 $${\text{TF}}_{\text{Fnr}} = \frac{{[O_{2} ]^{n} }}{{[O_{2} ]^{n} + K_{\text{Fnr}}^{n} }},$$

9 $${\text{TF}}_{\text{ArcA}} = \frac{{[Q]^{n} }}{{[Q]^{n} + K_{\text{ArcA}}^{n} }},$$

where *K*_Fnr_ and *K*_ArcA_ are the affinity constants and *n* is the negative Hill coefficient, and [*O*_2_] was defined by

10 $$[O_{2} ] = k_{{O_{2} }} [{\text{DO}}_{2} ],$$

where [DO_2_] is the dissolved oxygen concentration in the culture medium and $k_{{O_{2} }}$ is the model parameter. Based on the experimental observation using biosensor [40], we assumed that [*O*_2_] is lower than [DO_2_]. Figure 1 shows the effects of TFs on the primary metabolic pathways included in the present model. A “+” sign represents the case in which the transcription factor activates gene expression, whereas a “−” sign represents the case in which the TF represses gene expression. The gene name is written in brackets, where *npts* denotes the gene that codes for glucose transporters other than glucose-PTS, and *L_emp* denotes a hypothetical gene that codes for the lumped reactions through glyceraldehyde-3-phosphate dehydrogenase (GAPDH), phosphoglucokinase (Pgk), phosphoglucomutase (Pgm), and enolase (Eno). The DO concentration in the culture medium [DO_2_] was scaled from 0 to 100% and was defined as follows:

11 $${\text{DO}}\;[\% ] \equiv \frac{{\left[ {{\text{DO}}_{2} } \right]\;\;}}{{\left[ {{\text{DO}}_{2} } \right]*}} \times 100,$$

where [DO_2_]* represents the saturated DO concentration at 37 °C. Thus, 0 and 100% DO levels represent the absence of oxygen (anaerobic condition) and DO saturation at 37 °C, respectively.

### Model identification

Model parameters were adjusted so that the model can reproduce the experimental behavior of WT strain in the batch cultures under both micro-aerobic and aerobic conditions [41, 42], whereas other parameters, including the Michaelis–Menten and dissociation constants, were retained as those given in the references (Additional file 2). MATLAB (MathWorks) was used for all simulations. The ode15s was adopted as an ordinary differential equation solver.

## Results

### Experimental verification of the kinetic model

**Fig. 2:**
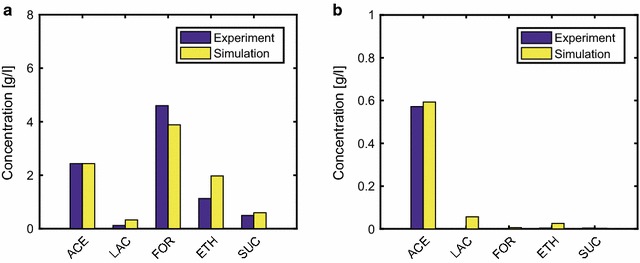
Comparison of the simulation results with experimental data for wild-type *E. coli.* Micro-aerobic (**a**) and aerobic (**b**) conditions. The experimental data for micro-aerobic and aerobic conditions refer to those reported by Zhu and Shimizu [42] and Toya et al. [41], respectively. The DO levels were set to 1 and 40% for the simulation under micro-aerobic and aerobic conditions, respectively, where these conditions are comparable to the experimental conditions. The simulation results show the product concentrations at the time points at which 10 g/l (micro-aerobic) and 4 g/l (aerobic) of glucose were depleted in the batch cultures

To verify the appropriateness of the kinetic model, the simulated extracellular concentrations of fermentation products were compared with the experimental data obtained from batch cultures under micro-aerobic (DO level = 1%) and aerobic (DO level = 40%) conditions [41, 42], as shown in Fig. 2. The concentrations of acetate, lactate, formate, ethanol, and succinate in the batch culture of the WT strain were plotted at the time points at which 10 g/l (micro-aerobic) and 4 g/l (aerobic) of glucose were depleted. The model reproduced most of the experimental product concentration under both micro-aerobic and aerobic conditions (Fig. 2). In addition, the model almost reproduced the experimental time courses of the WT strain under micro-aerobic (DO level = 9%) and aerobic conditions [43, 44], as shown in Additional file 3: Figure S1. The correlation coefficient between the measured [41–44] and simulated metabolite concentrations was 0.98 (*p* < 0.05), as shown in Additional file 3: Figure S2.

### Effect of DO level on the metabolic characteristics in the WT strain

**Fig. 3:**
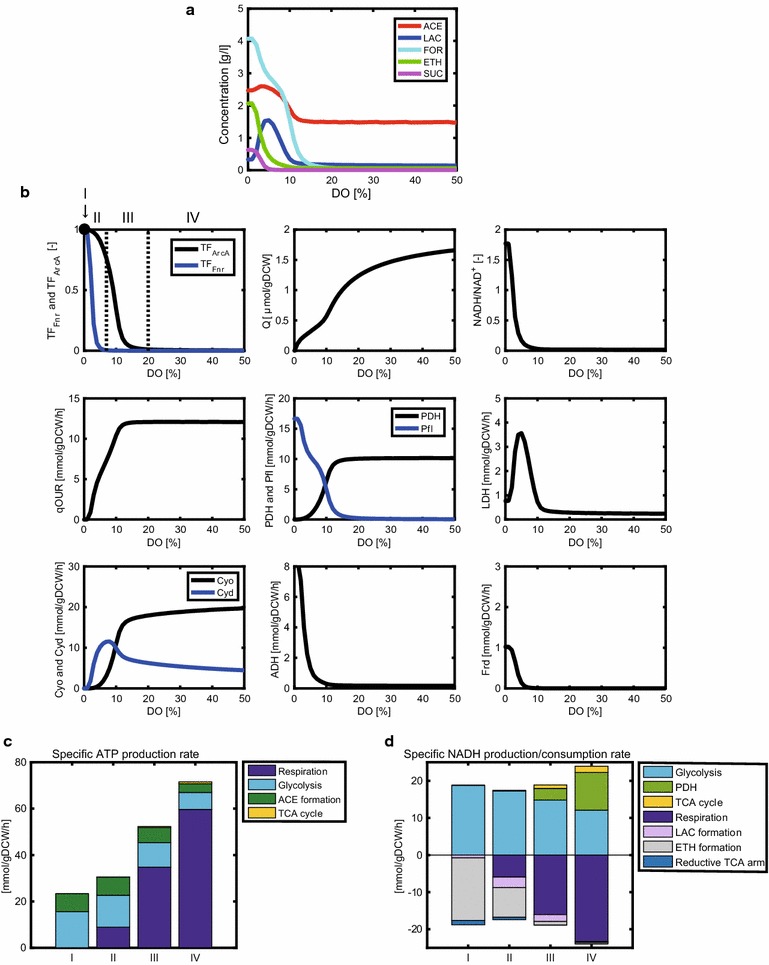
Simulation results of the metabolic changes with respect to DO level in wild-type *E. coli.* The changes in the metabolic (fermentation) products (**a**), and the activities of transcription factors, intracellular metabolites, and fluxes (**b**) with respect to DO level. The specific ATP production rate (**c**) and specific NADH production/consumption rates (**d**) are shown for the DO levels of 0, 3, 8, and 40% of air saturation, representatives of conditions I, II, III, and IV, respectively, where (I) anaerobic condition (DO = 0%); (II) micro-aerobic conditions under which both Fnr and ArcA are active; (III) micro-aerobic conditions under which ArcA is primarily active; and (IV) aerobic conditions under which neither Fnr nor ArcA is active. The simulation results show the product concentrations at the time point at which 10 g/l of glucose was depleted in a batch culture

Figure 3a shows the simulation result for a batch culture of the WT strain, in which the concentrations of acetate, lactate, formate, ethanol, and succinate were simulated at the time point at which 10 g/l of glucose was depleted. Acetate was the primary product at DO levels >15%. This acetate overflow was observed together with high CO_2_ production at a high growth rate in *E. coli* [45, 46], as shown in Additional file 3: Figure S3. The acetate concentration increased with decrease in the DO level (3–14%), as observed experimentally [24]. As the DO level decreased, the formate, ethanol, and succinate concentrations increased; the lactate concentration increased and then steeply decreased below 5% of DO level. At DO levels <2%, the lactate concentration was lower than that of the other products as experimentally observed [42, 47].

### Changes in the metabolism of the WT strain with respect to DO level

As shown in Fig. 3b, the changes in the concentrations of intracellular metabolites and fluxes were simulated with respect to DO level in the WT strain. The metabolic characteristics were evaluated by classifying the DO level into four categories: (I) anaerobic condition (DO = 0%) in which both Fnr and ArcA are active; (II) micro-aerobic conditions (0% < DO < 7%) in which both Fnr and ArcA are active; (III) micro-aerobic conditions (7% ≤ DO < 20%) in which ArcA is primarily active and Fnr is inactive; and (IV) aerobic conditions (DO ≥ 20%) in which neither Fnr nor ArcA is active. TF_Fnr_ and TF_ArcA_ in Fig. 3b were calculated by Eqs. 8 and 9, respectively. The change in the typical carbon metabolism is illustrated for these categories in Additional file 3: Figure S4.

The specific oxygen uptake rate (qOUR), which indicates the rate of oxygen consumption via Cyo and Cyd reactions, was simulated to be high under condition IV, whereas it decreased under conditions III, II, and I (Fig. 3b). As the DO level decreased, the Cyd flux increased and then decreased at <7% DO. This up and down behavior can be attributed to the activation of Cyd synthesis by ArcA under condition III, whereas Cyd synthesis was repressed by Fnr under conditions I and II (Fig. 1). The Cyo flux was simulated to be higher than the Cyd flux under condition IV, whereas the Cyd flux was more dominant than the Cyo flux under condition II. These simulation results are supported by the experimental fact that the affinity of Cyd to oxygen is higher than that of Cyo [29]. Since quinone is produced by Cyo and Cyd, quinone decreases with a decrease in DO level. This phosphorylates ArcB and then ArcA, resulting in the increase in the ArcA activity under conditions I, II, and III.

Among the enzymes associated with consumption of pyruvate, Pfl, pyruvate dehydrogenase (PDH), and LDH, play critical roles in determining the metabolite formation pattern. As the DO level decreased, the Pfl flux was simulated to increase under condition III because ArcA activated the Pfl reaction (Fig. 1). The Pfl flux was further enhanced by Fnr and ArcA under conditions I and II. In contrast, as the DO level decreased, the PDH flux decreased under condition III because ArcA represses the *aceE/F* genes that encode PDH (Fig. 1). The Pfl and PDH fluxes were both active under condition III, which was consistent with the experimental data [48]. The LDH flux exhibited an up and down behavior with respect to DO level. As the DO level decreased, the LDH flux increased under condition III, whereas it declined steeply under condition II.

The NADH/NAD^+^ ratio increased steeply with decreasing DO level under conditions I and II because NADH is hard to be consumed by the NADH dehydrogenases in the respiratory chain. This simulation result was consistent with the experimental data [49]. A high NADH/NAD^+^ ratio promoted the ADH reaction, which resulted in enhanced ethanol production under conditions I and II. Since Fnr activates the Frd flux (Fig. 1), succinate production was enhanced under conditions I and II.

To obtain a better understanding of the mechanisms by which ATP and NADH are produced or consumed under the categorized DO conditions examined, the specific production/consumption rates of ATP and NADH were simulated, as illustrated in Fig. 3c, d. DO levels of 0, 3, 8, and 40% were selected as the representatives of conditions I, II, III, and IV, respectively. The specific ATP production rate decreased in the order of conditions IV, III, II, and I (Fig. 3c). Additional file 3: Figure S5 indicates the relationship between the specific ATP production rate and the specific growth rate. Once the specific ATP production rate was calculated by Eq. 1, the specific growth rate was estimated by Eq. 3. This linear relationship between the specific ATP production rate and the specific growth rate held not only under aerobic conditions but also under micro-aerobic and anaerobic conditions with a correlation coefficient of 0.92 (*p* < 0.05), as experimentally observed [25, 37, 38, 41, 42, 47].

The DO level affected the specific ATP production rate (Fig. 3c). ATP was primarily synthesized by respiration under condition IV. By contrast, substrate-level phosphorylation by glycolysis and acetate formation became dominant under conditions I and II. NADH was consumed by the NADH dehydrogenases (Nuo and Ndh) in the respiratory chain under condition IV, whereas NADH was primarily consumed by ethanol formation under conditions I and II (Fig. 3d). This simulation result demonstrates that the ADH flux increased under conditions I and II due to a high NADH/NAD^+^ ratio (Fig. 3b). In fact, it was experimentally shown that ethanol is produced under micro-aerobic and anaerobic conditions [42, 47, 50]. The reaction of reductive TCA arm via malate dehydrogenase (MDH) consumed NADH under conditions I and II (Fig. 3d). The resultant fumarate/malate were supplied as the substrates for the reaction of Fnr-activated Frd (Fig. 3b), producing succinate (Fig. 3a). This simulation result was consistent with the experimental observation [47, 50]. NADH was produced by glycolysis, the PDH reaction, and the TCA cycle under condition IV (Fig. 3d), whereas NADH production by the PDH flux and TCA cycle declined significantly under conditions I and II because ArcA represses the PDH flux and both ArcA and Fnr repress the TCA cycle.

Additional file 3: Figure S3A shows the carbon balances of the extracellular products, CO_2_, and biomass at different DO levels (conditions I, II, III, and IV) in the WT strain. The metabolic modes changed significantly depending on DO level. Most of glucose was converted to biomass, CO_2_, and acetate under condition IV. On the other hand, biomass and CO_2_ production were decreased under condition I.

### Prediction of the metabolic characteristics of an *fnr*-knockout mutant

**Fig. 4:**
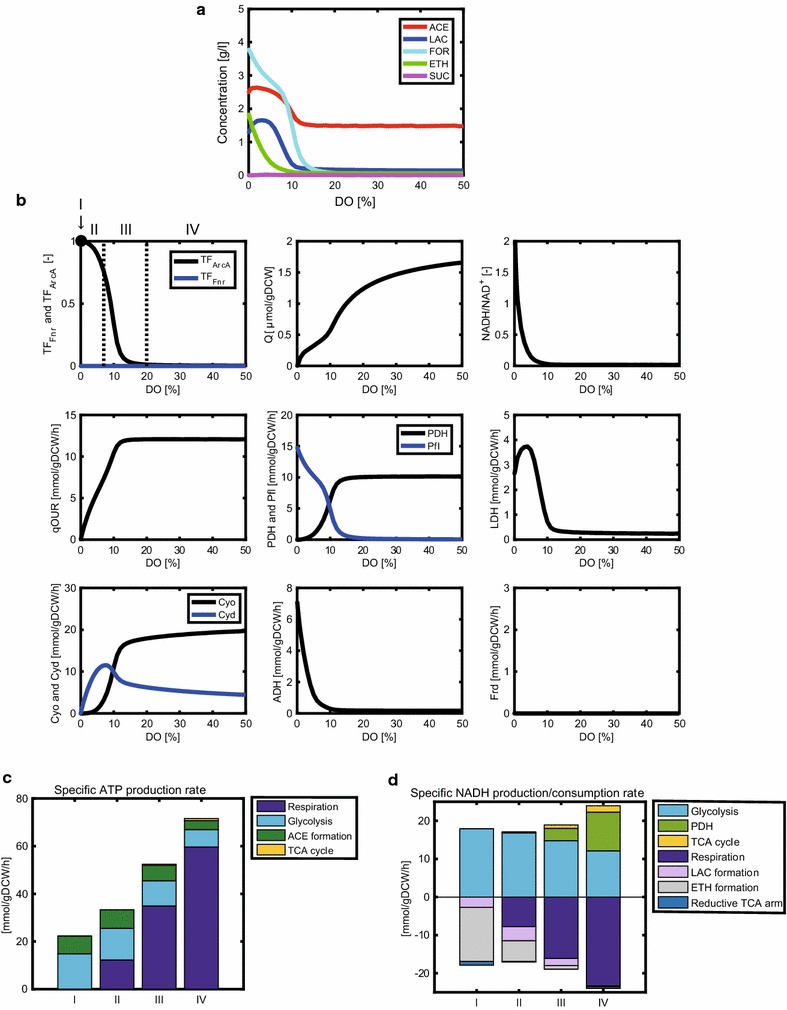
Simulation results of the metabolic changes with respect to DO level in *fnr*-knockout mutant. The changes in the metabolic (fermentation) products (**a**), and the activities of transcription factors, intracellular metabolites, and fluxes (**b**) with respect to DO level. The specific ATP production rate (**c**) and specific NADH production/consumption rates (**d**) are shown for the DO levels of 0, 3, 8, and 40% of air saturation, representatives of conditions I, II, III, and IV, respectively. The simulation results show the product concentrations at the time point at which 10 g/l of glucose was depleted in a batch culture

As Fnr and ArcA play critical roles in redox regulation at low DO levels, it is of interest to predict the effect of *fnr* or *arcA* gene knockout on the primary metabolism. Figure 4a shows the simulation results of an *fnr*-knockout mutant, in which the concentrations of acetate, lactate, formate, ethanol, and succinate were simulated at the time point at which 10 g/l of glucose was depleted. As compared to the WT strain (Fig. 3a), succinate was rarely produced at any DO level due to little activity of Frd caused by a lack of Fnr. As DO level decreased, lactate increased, peaking at 3% DO, and then slightly decreased. The lactate production in the *fnr*-knockout mutant was more enhanced than the WT strain at very low DO levels, which was supported by the experimental data [51].

Figure 4b shows the effect of DO level on the intracellular metabolic fluxes, redox status, and transcriptional activities. The Pfl flux was predicted to be lower in the *fnr*-knockout mutant than in the WT strain (Fig. 3b) under conditions I and II. The LDH flux increased under condition III and slightly decreased under conditions I and II in the *fnr*-knockout mutant, but it was higher than that of the WT strain (Fig. 3b). As the DO decreased, the NADH/NAD^+^ ratio increased under conditions III, II, and I, which resulted in the increased ADH flux, while the Frd flux was zero due to a lack of Fnr.

The simulated specific production/consumption rates of ATP and NADH are shown in Fig. 4c, d. The profiles of the specific ATP production rates of the *fnr*-knockout mutant were almost the same as those of the WT strain (Fig. 3c), whereas the specific NADH consumption rate in the lactate and ethanol formation through LDH and ADH and the respiratory pathway somewhat differed from that of the WT strain under condition I (Figs. 3d, 4d). The NADH consumption rate through LDH in the *fnr*-knockout mutant was higher than that of the WT strain (as discussed later), whereas the NADH consumption rate through ADH was lower than that of the WT strain. As compared with the WT strain, the NADH consumption rate by the NADH dehydrogenases in the respiratory chain increased under condition II because the lack of Fnr de-repressed the NADH dehydrogenase reactions.

Additional file 3: Figure S3B shows the carbon balances of the metabolic products including CO_2_ and biomass in the *fnr*-knockout mutant at different DO levels. The carbon balances differed between the WT strain and the *fnr*-knockout mutant under conditions I and II (Additional file 3: Figure S3A, B). More glucose carbon was converted into lactate in the *fnr*-knockout mutant than in the WT strain.

### Prediction of the metabolic characteristics of an *arcA*-knockout mutant

**Fig. 5:**
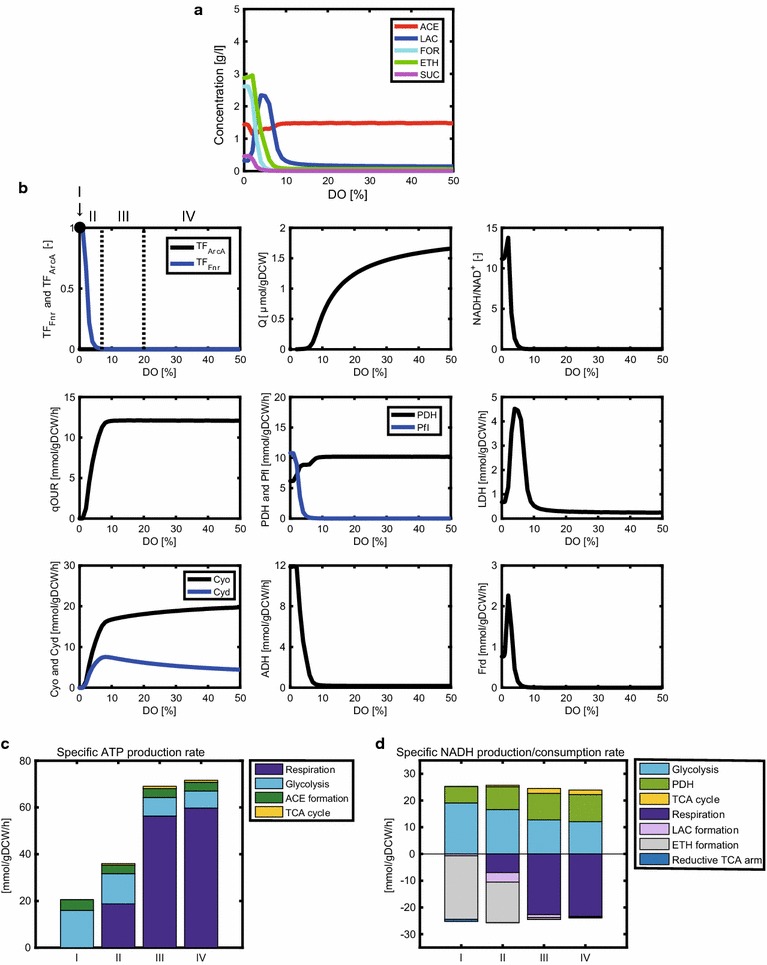
Simulation results of the metabolic changes with respect to DO level in *arcA*-knockout mutant. The changes in the metabolic (fermentation) products (**a**), and the activities of transcription factors, intracellular metabolites, and fluxes (**b**) with respect to DO level. The specific ATP production rate (**c**) and specific NADH production/consumption rates (**d**) are shown for the DO levels of 0, 3, 8, and 40% of air saturation, representatives for the conditions I, II, III, and IV, respectively. The simulation results show the product concentrations at the time point at which 10 g/l of glucose was depleted in a batch culture

As shown in Fig. 5a, the model predicted the changes in metabolic products with respect to DO level in an *arcA*-knockout mutant. Acetate production decreased slightly from 10 to 2% DO, as experimentally observed [52]. Ethanol production was predicted to be higher in the *arcA*-knockout mutant than in the WT strain and *fnr*-knockout mutant at DO <6%, which was consistent with the experimental data under micro-aerobic conditions [53, 54]. Under anaerobic condition, ethanol production was also predicted to be higher in the *arcA*-knockout mutant than in the other strains, while the ethanol production in the *arcA*-knockout mutant was reduced in the experiment [53]. This discrepancy will be discussed later. As the DO level decreased, lactate increased, peaking at 4% DO, and then decreased (Fig. 5a). The maximum concentration of lactate produced by the *arcA*-knockout mutant was higher than those of the WT strain and *fnr*-knockout mutant, as experimentally observed [53].

Figure 5b shows the effect of the DO level on the intracellular metabolic fluxes, redox status, and transcriptional activities. The Cyd flux was lower than that of the WT strain under conditions III and II (Figs. 3b, 5b), as experimentally observed [55]. As the DO level decreased under conditions III and II, the PDH flux slightly decreased. The decrease in the PDH flux was small compared to that of the WT strain and the *fnr*-knockout mutant (Figs. 3b, 4b, 5b) because the PDH flux is not repressed in the *arcA*-knockout mutant. The NADH/NAD^+^ ratio in the *arcA*-knockout mutant was higher than that of the WT strain and the *fnr*-knockout mutant under condition II (Figs. 3b, 4b, 5b), as experimentally observed [53]. As DO level decreased, the NADH/NAD^+^ ratio increased and then declined slightly as experimentally observed [53]. The simulated NADH/NAD^+^ ratio of the *arcA*-knockout mutant was higher than its experimental ratio, although the simulated NADH/NAD^+^ ratios of the WT strain and the *fnr*-knockout mutant were relatively consistent with their experimental ratios. While the activities of the PDH, citrate synthase (CS), and isocitrate dehydrogenase (ICDH) enzymes are allosterically inhibited by NADH to suppress an excess production of NADH [56, 57], the present model did not implement such allosteric inhibitions. The neglect of the allosteric inhibitions relatively reproduced the NADH/NAD^+^ ratios of the WT strain and the *fnr*-knockout mutant because their NADH level was not so high as that of the *arcA*-knockout mutant, but would overestimate the NADH/NAD^+^ ratio of the *arcA*-knockout mutant. As the DO level decreased, the Frd flux steeply increased and then declined (Fig. 5b). The simulation result of the Frd flux showed the similar trend as that of the NADH/NAD^+^ ratio due to the fact that NADH is oxidized at MDH with Frd.

The specific production/consumption rates of ATP and NADH were simulated as shown in Fig. 5c, d. The specific ATP production rate increased in the *arcA*-knockout mutant under condition III as compared with the WT strain (Figs. 3c, 5c) because Cyo is activated in the *arcA*-knockout mutant (Fig. 1). For this, the qOUR for the *arcA*-knockout mutant was higher than that of the WT strain under condition III (Figs. 3b, 5b), as experimentally observed [32]. The specific NADH production rate in the TCA cycle was slightly higher than that of the WT strain under condition III (Figs. 3d, 5d), as experimentally observed [32], because the TCA cycle is not repressed in this mutant. The specific NADH consumption rate through ethanol formation (by ALDH and ADH) was higher than that of the WT strain under conditions I and II (Figs. 3d, 5d).

Additional file 3: Figure S3C shows the carbon balances of the metabolic products including CO_2_ and biomass in the *arcA*-knockout mutant at different DO levels. More glucose carbon was converted to biomass, and more CO_2_ was produced in the *arcA*-knockout mutant than the WT strain due to de-repression of the PDH and TCA cycle under condition III (Additional file 3: Figure S3A, C).

### Rational design of a method for lactate production by a *pfl*-knockout mutant

**Fig. 6:**
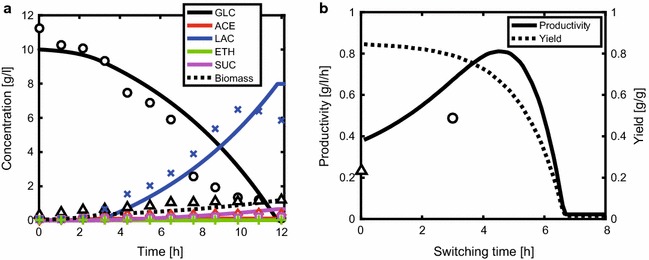
Batch cultivation of *pfl*-knockout mutant in the dual-phase cultivation starting with aerobic cultivation followed by micro-aerobic cultivation. The DO levels of 40 and 1% were set for the aerobic and micro-aerobic conditions, respectively. 10 g/l glucose was used as a carbon source. **a** Time course data of extracellular product and biomass cultured for 3 h under aerobic condition followed by micro-aerobic condition, where the lines show simulation results and the symbols represent experimental data [44]: *open circle* glucose; *open diamond* acetate; *multiplication sign* lactate; *full width plus sign* ethanol; *open square* succinate; *open up*-*pointing triangle* biomass. **b** Effect of the switching time on the yield and productivity. *Yield* represents g of lactate/g of glucose consumed. *Productivity* represents g/l of lactate concentration/h of cultivation time. *The lines* represent the simulation results and the *symbols* represent the experimental data of the productivity (*open up*-*pointing triangle* Liu et al. [58]; *open circle* Zhu and Shimizu [44])

The present model was utilized for the rational design of microbial cellular factories and optimization of target metabolite production. While a *pfl*-knockout mutant was experimentally reported to exclusively produce lactate [44], the effect of the DO level on the energy generation, biomass formation, and productivity has been rarely investigated. Simulated time course data of a batch culture of the *pfl*-knockout mutant reasonably predicted the experimental data (Fig. 6a) [44]. Here, we considered operation strategies for the efficient production of lactate by the *pfl*-knockout mutant.

Dual-phase cultivation was designed to enhance the target metabolite production, starting with an aerobic cultivation to promote the cell growth, followed by an anaerobic or micro-aerobic condition to facilitate the target metabolite production. The switching time when the culture condition is changed from aerobic to micro-aerobic condition is generally a key parameter for enhanced productivity. The effect of the switching time on lactate yield (g of product/g of substrate consumed) and productivity (g/l of product concentration/h of cultivation time) was simulated for the *pfl*-knockout mutant when 10 g/l glucose was supplied as a carbon source (Fig. 6b). The DO levels of 40 and 1% were set to the aerobic and micro-aerobic conditions, respectively. The symbols in Fig. 6b represent the productivity of lactate obtained from the experiments [44, 58]. As expected, the yield was the highest when the cells were cultured consistently under the micro-aerobic condition, although the productivity was low. The productivity was improved to 0.81 g/l/h (at a switching time of 4.5 h) by the dual-phase cultivation, as compared to 0.38 g/l/h under the micro-aerobic condition throughout the cultivation (Fig. 6b).

## Discussion

### Advantages of the proposed kinetic model

There are several modeling approaches to simulate the fermentation characteristics. Khodayari et al. [59] simulated the succinate overproduction by *E. coli* under both aerobic and anaerobic conditions using the kinetic model-based k-OptForce method with ensemble modeling approach and parameterization based on the data obtained from multiple mutant strains. Their model was able to predict the metabolism that improves the succinate yield under aerobic condition but failed to predict it under anaerobic condition. It is essential to predict the dynamics of the cell growth and metabolite production over a broad range of DO levels and to understand the metabolic regulation mechanisms for the rational design of useful metabolite production. To meet these requirements, we have developed a kinetic model that implements redox regulation by Fnr and ArcA into central carbon metabolism [15, 20]. An advantage in the proposed model is to accurately simulate metabolisms under anaerobic, micro-aerobic, and aerobic conditions.

The model was constructed and verified using available experimental data for the WT strain [24, 29, 41–44, 47–51, 54]. The model-predicted behaviors were validated by the experimental data of the *fnr*-knockout mutant [51], the *arcA*-knockout mutant [32, 52, 53, 55, 60], and the *pfl*-knockout mutant [44].

To achieve efficient production of a target metabolite, the dual-phase cultivation method was investigated to improve the lactate production using the *pfl*-knockout mutant. This investigation revealed the importance of the optimal switching time from aerobic to micro-aerobic conditions to maximize the productivity (Fig. 6b). In addition, the trade-off between yield and productivity must be considered in practice, because the yield decreases with increased duration of the aerobic period (Fig. 6b).

### Regulation mechanisms underlying the metabolic changes in response to DO level

In the simulation of the WT strain, the LDH flux exhibited up and down changes with respect to DO level (Fig. 3b). The LDH flux increased more under condition III than under condition IV. Under condition III, ArcA repressed the PDH flux while increasing the Pfl flux. Although the total flux from pyruvate to acetyl-CoA (PDH flux + Pfl flux) was almost the same between under conditions III and IV, the increased NADH/NAD^+^ ratio increased the LDH flux under condition III. On the other hand, the LDH flux decreased under condition II because pyruvate, the substrate of the LDH reaction, was consumed by the Fnr-enhanced Pfl reaction.

Lactate production increased in the *fnr*-knockout mutant under conditions I and II (Figs. 3a, 4a) as compared with the WT strain, as experimentally observed [51]. Since the Pfl flux was reduced in the *fnr*-knockout mutant under conditions I and II (Figs. 3b, 4b), the total flux from pyruvate to acetyl-CoA was also reduced as compared to the WT strain, resulting in the accumulation of pyruvate. Pyruvate was converted to lactate, accompanied by NADH consumption. On the other hand, the *arcA*-knockout mutant also exhibited higher lactate production than the WT strain around 4% DO under condition II (Figs. 3a, 5a) because the total flux from pyruvate to acetyl-CoA was reduced as compared to the WT strain as experimentally observed [60].

At lower oxygen levels under conditions I and II, the *arcA*-knockout mutant exhibited a marked increase in ethanol production (Figs. 3a, 5a). Although the total flux from pyruvate to acetyl-CoA was almost the same as that of the WT strain under conditions I and II, the NADH/NAD^+^ ratio in the *arcA*-knockout mutant was much higher than those in the WT strain and the *fnr*-knockout mutant due to the high flux of PDH (Figs. 3b, 4b, 5b). This resulted in enhanced ethanol production. These simulation results were consistent with the experimental observation except for condition I (anaerobic condition) [53]. While the simulated ethanol production flux of the *arcA*-knockout mutant was higher than that of the WT strain, the experimental ethanol production flux in the *arcA*-knockout mutant was comparable to that in the WT strain under anaerobic condition. The discrepancy in the ethanol production under anaerobic condition would be due to the overestimation of the NADH/NAD^+^ ratio in the *arcA*-knockout mutant. This overestimation results from the fact that the simulated reductive pathway flux through Ppc-MDH/Fum-Frd in the *arcA*-knockout mutant was lower than the experimental flux under anaerobic condition. Such underestimation of the reductive flux may be caused by the neglect of some effectors [61, 62] on the Ppc reaction responsible for the MDH and Frd fluxes. The present model includes the effect of FBP on the Ppc activity, but did not include the effects of acetyl-CoA, malate, and aspartate.

### Toward virtual metabolism

Synthetic biology aims to understand the mechanisms governing the dynamic behaviors of biochemical networks in response to environmental stresses or genetic variations and facilitate the rational design or engineering of cells at the gene-regulation level. Synthetic biology approaches consist of the construction of a rigorously defined biochemical network map, development of mathematical models, experimental validation of these models, and analysis and rational design of biological systems, ultimately leading to computer-aided design of cells [63–65]. The proposed kinetic model was constructed according to this synthetic approach to provide a platform for the rational design of biofuel and biochemical production by *E. coli* and for further modeling efforts, including extension to amino acid, nucleotide, lipid, and polysaccharide metabolisms, as well as cell physiology. A comprehensive dynamic model, called ‘virtual *E. coli*,’ is expected to reproduce the complex dynamics of a series of genetic mutants under different conditions, such as consumption of multiple sugars, nitrogen, and phosphate starvation, osmotic pressure, and changes in pH. In addition, the kinetic model of the *E. coli* central carbon metabolism would be a feasible reference model for constructing the kinetic models of a variety of microbes, because central carbon metabolisms are relatively conserved across them.

On the other hand, another characteristic of microbes is their metabolic variety due to evolution under various growth conditions on earth. For example, yeast produces ethanol via pyruvate decarboxylase (PDC) and ADH, *Clostridia* employs acetone–butanol–ethanol (ABE) pathway, and *Zymomonas* has the Entner–Doudoroff (ED) pathway. Since the detailed metabolic pathways depend on the microbes, it is essential to take into account their differences to construct their kinetic models, while using the *E. coli* kinetic model as a reference model.

## Conclusions

It is quite important to understand metabolic regulation mechanisms from both scientific and engineering points of view. In particular, redox regulation in response to oxygen limitation is critically important in the practical production of biofuel and biochemical compounds. Therefore, we developed a kinetic model with enzymatic and transcriptional regulations to predict the dynamics of metabolism at different DO levels. Transcription factor activities, metabolite concentrations, and fluxes of the WT strain and *fnr*- and *arcA*-knockout mutants were simulated to validate the model. Using this kinetic model, a rational operation strategy for the *pfl*-knockout mutant was designed to enhance lactate production. A dual-phase strategy was considered that involves initial cultivation under aerobic condition to enhance the cell growth rate, with subsequent cultivation under anaerobic or micro-aerobic condition to enhance the lactate production.

## Abbreviations

### Primary metabolic pathway and transport system

*EI* enzyme I, *EIIA* enzyme IIA, *ED* pathway, Entner–Doudoroff pathway, *HPr* histidine-phosphorylatable protein, *PP* pathway, pentose phosphate pathway, *PTS* phosphotransferase system, *TCA cycle* tricarboxylic acid cycle.

### Metabolites

*ACAL* acetaldehyde, *AcCoA* acetyl-CoA, *CIT* citrate, *DHAP* dihydroxy acetone phosphate, *E4P* erythrose-4-phosphate, *ETH* ethanol, *FBP* fructose-1,6-bisphosphate, *FOR* formate, *F6P* fructose-6-phosphate, *FUM* fumarate, *G6P* glucose-6-phosphate, *GAP* glyceraldehyde-3-phosphate, *GLC* glucose, *GOX* glyoxylate, *ICI* isocitrate, *αKG* α-ketoglutarate, *LAC* lactate, *MAL* malate, *OAA* oxaloacetate, *PEP* phosphoenol pyruvate, *6PG* 6-phosphogluconate, *6PGL* 6-phosphogluconolactone, *PYR* pyruvate, *Q* quinone, *QH*_*2*_ quinol, *R5P* ribose-5-phosphate, *RU5P* ribulose-5-phosphate, *S7P* sedoheptulose-7-phosphate, *SUC* succinate.

### Enzymes

*Ack* acetate kinase, *Acs* acetyl coenzyme A synthetase, *ADH* alcohol dehydrogenase, *ALDH* acetaldehyde dehydrogenase, *Cya* adenylate cyclase, *Cyd* cytochrome *bd*, *Cyo* cytochrome *bo*, *CS* citrate synthase, *Eno* enolase, *Fba* fructose-1,6-bisphosphate aldolase, *Fbp* fructose bisphosphatase, *Frd* fumarate reductase, *Fum* fumarase, *G6PDH* glucose-6-phosphate dehydrogenase, *GAPDH* glyceraldehyde-3-phosphate dehydrogenase, *Glk* glucokinase, *ICDH* isocitrate dehydrogenase, *Icl* isocitrate lyase, *αKGDH* α-ketoglutarate dehydrogenase, *LDH* lactate dehydrogenase, *MDH* malate dehydrogenase, *Mez* malic enzyme, *MS* malate synthase, *Ndh* NADH dehydrogenase-II, *Nuo* NADH dehydrogenase-I, *Pck* phosphoenolpyruvate carboxykinase, *PDH* pyruvate dehydrogenase, *Pfk* phosphofructokinase, *Pfl* pyruvate formate-lyase, *PGDH* 6-phosphogluconate dehydrogenase, *Pgk* phosphoglucokinase, *Pgm* phosphoglucomutase, *Ppc* phosphoenolpyruvate carboxylase, *Pps* phosphoenolpyruvate synthase, *Pta* phosphotransacetylase, *Pyk* pyruvate kinase, *Rpe* ribulose phosphate epimerase, *Rpi* ribose phosphate isomerase, *SDH* succinate dehydrogenase, *Tal* transaldolase, *TktA* transketolase I, *TktB* transketolase II.

## Additional files

**Additional file 1.** Detailed model equations.

**Additional file 2.** Model parameters.

**Additional file 3.** Additional results; including additional figures S1–S5.
