# Supplementary material for: Modeling and simulation of the redox regulation of the metabolism in Escherichia coli at different oxygen concentrations
Source: Biotechnol Biofuels. 2017 Jul 14;10:183. doi: 10.1186/s13068-017-0867-0 (PMC5512849; doi:10.1186/s13068-017-0867-0)
Supplement: Supplementary file 1 — Additional file 1. Detailed model equations. [file 13068_2017_867_MOESM1_ESM.doc]

**Additional file 1**

**Mass balance equations**

Referring to Fig. 1 in the main text, the mass balance equations are expressed as follows:

| **Fermentative variables** |  |
| --- | --- |
| $\frac{d[X]}{dt}=\mu[X]$ | (S1) |
| $\frac{d[GLC]}{dt}=-v_{GLC,uptake}$ | (S2) |
| $\frac{d[ACT]}{dt}=v_{ACT,excrete}-v_{ACT,uptake}$ | (S3a) |
| $\frac{d[LAC]}{dt}=v_{LAC,excrete}$ | (S3b) |
| $\frac{d[FOR]}{dt}=v_{FOR,excrete}$ | (S3c) |
| $\frac{d[ETH]}{dt}=v_{ETH,excrete}$ | (S3d) |
| $\frac{d[SUC]}{dt}=v_{SUC,excrete}$ | (S3e) |
| **Intracellular metabolites** |  |
| $\frac{d[GLC^{in}]}{dt}=v_{NPTS}-v_{Glk}-\mu[GLC^{in}]$ | (S4a) |
| $\begin{matrix} \frac{d[G6P/F6P]}{dt}=v_{PTS4}+v_{Fbp}+v_{TktB}+v_{Tal}+v_{Glk} \\ -v_{Pfk}-v_{G6PDH}-v_{Bio,G6P}-\mu[G6P] \end{matrix}$ | (S4b) |
| $\frac{d[FBP]}{dt}=v_{Pfk}-v_{Fba}-v_{Fbp}-\mu[FBP]$ | (S4c) |
| $\begin{matrix} \frac{d[GAP/DHAP]}{dt}=2v_{Fba}+v_{TktA}+v_{TktB} \\ -v_{L\_Emp}-v_{Tal}-v_{Bio,GAP}-\mu[GAP/DHAP] \end{matrix}$ | (S4d) |
| $\frac{d[PEP]}{dt}=v_{L\_Emp}+v_{Pck}+v_{Pps}-v_{Pyk}-v_{Ppc}-v_{PTS1}-v_{Bio,PEP}-\mu[PEP]$ | (S4e) |
| $\frac{d[PYR]}{dt}=v_{Pyk}+v_{Mez}+v_{PTS1}-v_{PDH}-v_{Pps}-v_{LDH}-v_{Pfl}-v_{Bio,PYR}-\mu[PYR]$ | (S4f) |
| $\begin{matrix} \frac{d[AcCoA]}{dt}=v_{PDH}+v_{Acs}+v_{Pfl} \\ -v_{CS}-v_{PTACK}-v_{MS}-v_{ALDH}-v_{Bio,AcCoA}-\mu[AcCoA] \end{matrix}$ | (S4g) |
| $\frac{d[ACAL]}{dt}=v_{ALDH}-v_{ADH}$ | (S4h) |
| $\frac{d[PGL/6PG]}{dt}=v_{G6PDH}-v_{PGDH}-\mu[PGL/6PG]$ | (S4i) |
| $\frac{d[RU5P]}{dt}=v_{PGDH}-v_{Rpe}-v_{Rpi}-\mu[RU5P]$ | (S4j) |
| $\frac{d[R5P]}{dt}=v_{Rpi}-v_{TktA}-v_{Bio,R5P}-\mu[R5P]$ | (S4k) |
| $\frac{d[X5P]}{dt}=v_{Rpe}-v_{TktA}-v_{TktB}-\mu[X5P]$ | (S4l) |
| $\frac{d[S7P]}{dt}=v_{TktA}-v_{Tal}-\mu[S7P]$ | (S4m) |
| $\frac{d[E4P]}{dt}=v_{Tal}-v_{TktB}-v_{Bio,E4P}-\mu[E4P]$ | (S4n) |
| $\frac{d[CIT/ICIT]}{dt}=v_{CS}-v_{ICDH}-v_{Icl}-\mu[CIT/ICIT]$ | (S4o) |
| $\frac{d[\alpha KG]}{dt}=v_{ICDH}-v_{\alpha KGDH}-v_{Bio,\alpha KG}-\mu[\alpha KG]$ | (S4p) |
| $\frac{d[SUC^{in}]}{dt}=v_{\alpha KGDH}+v_{Icl}+v_{Frd}-v_{SDH}-v_{SUC_{trans}}-\mu[SUC^{in}]$ | (S4q) |
| $\frac{d[MAL]}{dt}=v_{SDH}+v_{MS}-v_{MDH}-v_{Frd}-v_{Mez}-\mu[MAL]$ | (S4r) |
| $\frac{d[OAA]}{dt}=v_{MDH}+v_{Ppc}-v_{CS}-v_{Pck}-v_{Bio,OAA}-\mu[OAA]$ | (S4s) |
| $\frac{d[GOX]}{dt}=v_{Icl}-v_{MS}-\mu[GOX]$ | (S4t) |
| **PTS proteins, cAMP, and transcription factors** |  |
| $\frac{d[EIIA]}{dt}=v_{PTS4}-v_{PTS1}$ | (S5a) |
| $\frac{d[EIIA\sim P]}{dt}=v_{PTS1}-v_{PTS4}$ | (S5b) |
| $\frac{d[cAMP]}{dt}=v_{Cya}-v_{cAMP\deg r}-\mu[cAMP]$ | (S6) |
| $\frac{d[cAMP-Crp]}{dt}=v_{cAMP-Crp}-\mu[cAMP-Crp]$ | (S7a) |
| $\frac{d[Cra-FBP]}{dt}=v_{Cra-FBP}-\mu[Cra-FBP]$ | (S7b) |
| $\frac{d[PdhR-PYR]}{dt}=v_{PdhR-PYR}-\mu[PdhR-PYR]$ | (S7c) |
| **Quinone (Q)/quinol (QH_2_) redox couple and NADH** |  |
| $\frac{d[QH_{2}]}{dt}=v_{Nuo}+v_{Ndh}+v_{Syn,QH_{2}}+v_{SDH}-v_{Cyo}-v_{Cyd}-\mu[QH_{2}]$ | (S8a) |
| $\frac{d[Q]}{dt}=v_{Cyo}+v_{Cyd}-v_{Nuo}-v_{Ndh}-v_{SDH}-\mu[Q]$ | (S8b) |
| $\begin{matrix} \frac{d[NADH]}{dt}=v_{Emp}+v_{PDH}+v_{\alpha KGDH}+v_{MDH} \\ -v_{LDH}-v_{ALDH}-v_{ADH}-v_{Nuo}-v_{Ndh}-\mu[NADH] \end{matrix}$ | (S9) |

where $\mu$ is the specific growth rate, and $[X]$ is the biomass concentration. The suffix “$Bio$” of means the biosynthetic pathway. $v_{\bullet}$ is the function of substrate(s), product(s), or metabolite(s) which allosterically affect the reaction rate as well as the function of transcription factors based on the relationship as given in Fig. 1 in the main text. Moreover, the total concentrations for transcription factor and NADH are expressed as follows:

| $[Crp]_{total}=[Crp]+[cAMP-Crp]$ | (S10a) |
| --- | --- |
| $[Cra]_{total}=[Cra]+[Cra-FBP]$ | (S10b) |
| $[PdhR]_{total}=[PdhR]+[PdhR-PYR]$ | (S10c) |
| $[NA*]_{total}=[NADH]+[NAD]$ | (S10d) |

**Kinetic models**

**Modeling for PTS**

For the glucose phosphorylation step of PTS, a reversible ping-pong mechanism and second-order rate law was considered (Kremling et al., 2001). Here, we assumed EI and HPr to be in equilibrium, and considered only EIIA and phosphorylated EIIA, EIIA-P in relation to PEP, PYR, Glc and G6P (Eqs. S5a and S5b). The resulting $v_{PTS1}$ and $v_{PTS4}$ may be expressed as (Kotte et al., 2010)

| $v_{PTS1}=k_{1}[PEP][EIIA]-k_{-1}[PYR][EIIA\sim P]$ | (S11a) |
| --- | --- |
| $v_{PTS4}=\frac{v_{PTS4}^{\max}\left[EIIA\sim P\right] [Glc]}{\left(K_{EIIA\sim P}+\left[EIIA\sim P\right]\right)\left(K_{Glc}+\left[Glc\right]\right)}$ | (S11b) |

**Modeling for glucose uptake by non-PTS pathway**

The glucose uptake via non-PTS transporters such as MglBAC and GalP is followed by the phosphorylation by Glk. Here, all of these transport systems other than glucose-PTS were assumed to be represented by NPTS, and $v_{NPTS}$ and $v_{Glk}$ were expressed as (Bettenbrock et al., 2006)

| $v_{NPTS}=\frac{v_{NPTS}^{\max}[GLC]}{K_{S}+\left(1+\frac{\left[EIIA\right]}{K_{I}}\right)\left[GLC\right]}$ | (S12a) |
| --- | --- |
| $v_{Glk}=\frac{v_{Glk}^{\max}[GLC^{in}]}{\left(1+\frac{\left[G6P/F6P\right]}{K_{I}}\right)\left(K_{S}+[GLC^{in}]\right)}$ | (S12b) |

**Modeling for cAMP**

Cya (adenylate cyclase) generates cAMP, and its transcription is activated by EIIA-P. The cAMP generation ($v_{Cya}$) and degradation ($v_{cAMP\deg r}$) were expressed as (Kremling et al., 2001; Kotte et al., 2010)

| $v_{Cya}=\frac{v_{Cya}^{\max}\left[EIIA\sim P\right]}{\left[EIIA\sim P\right]+K_{EIIA\sim P}}$ | (S13a) |
| --- | --- |
| $v_{cAMP\deg r}=\frac{v_{Degr}^{\max}[cAMP]}{[cAMP]+K_{cAMP}}$ | (S13b) |

**Modeling for glycolysis**

Kinetic model equations has been proposed by several researchers in the past (Chassagnole et al., 2002; Kotte et al., 2010). Here we considered to lump Pgi and Pfk reactions together, where $v_{Pfk}$ was expressed as a function of its substrate G6P and allosteric inhibition term by PEP such as (Kotte et al., 2010)

| $v_{Pfk}=\frac{v_{Pfk}^{\max}\frac{[G6P/F6P]}{K_{G6P/F6P}}\left(1+\frac{[G6P/F6P]}{K_{G6P/F6P}}\right)^{n-1}}{\left(1+\frac{[G6P/F6P]}{K_{G6P/F6P}}\right)^{n}+L\left(1+\frac{[PEP]}{K_{PEP}}\right)^{n}}$ | (S14a) |
| --- | --- |

The reaction rate through Fba was expressed as (Kotte et al., 2010)

| $v_{Fba}=\frac{v_{Fba}^{\max,f}\frac{[FBP]}{K_{FBP}}- v_{Fba}^{\max,r}\frac{[GAP/DHAP]}{K_{GAP/DHAP}}}{1+\frac{[FBP]}{K_{FBP}}+\frac{[GAP/DHAP]}{K_{GAP/DHAP}}}$ | (S14b) |
| --- | --- |

Then, the reactions through GAPDH, Pgk, Pgm, and Eno were lumped together as (Kotte et al., 2010)

| $v_{L\_Emp}=\frac{v_{L\_Emp}^{\max,f} \frac{[GAP/DHAP]}{K_{GAP/DHAP}}-v_{L\_Emp}^{\max,r}\frac{[PEP]}{K_{PEP}}}{1+\frac{[GAP/DHAP]}{K_{GAP/DHAP}}+\frac{[PEP]}{K_{PEP}}}$ | (S14c) |
| --- | --- |

The reaction rate for Pyk was expressed as (Kotte et al., 2010)

| $v_{Pyk}=\frac{ v_{Pyk}^{\max}\frac{[PEP]}{K_{PEP}}\left(1+\frac{[PEP]}{K_{PEP}}\right)^{n-1}}{\left(1+\frac{[PEP]}{K_{PEP}}\right)^{n}+L\left(1+\frac{[FBP]}{K_{FBP}}\right)^{-n}}$ | (S14d) |
| --- | --- |

where this is a function of its substrate PEP and the allosteric activator FBP.

**Modeling for PP pathways**

The kinetic model equations have also been proposed by several researchers (Chassagnole et al., 2002). Here the oxidative PP pathway reactions such as $v_{G6PDH}$ and $v_{PGDH}$ were expressed as a growth rate dependent (Wolf et al. 1979). Based on the similar equations as (Chassagnole et al., 2002)

| $v_{G6PDH}=\frac{v_{G6PDH}^{\max} [G6P][NADP]}{\left([G6P]+K_{G6P}\right)\left(1+\frac{[NADPH]}{K_{NADPH\_G6Pinh}}\right)\left(K_{NADP}\left(1+\frac{[NADPH]}{K_{NADPH\_NADPinh}}\right)+[NADP]\right)}$ | (S15a) |
| --- | --- |
| $v_{PGDH}=\frac{v_{PGDH}^{\max} [6PG][NADP]}{\left([6PG]+K_{6PG}\right)\left([NADP]+K_{NADP}\left(1+\frac{[NADPH]}{K_{NADPHinh}}\right)\left(1+\frac{[ATP]}{K_{ATPinh}}\right)\right)}$ | (S15b) |

where $v_{G6PDH}^{\max}=k_{G6PDH}\cdot\mu\cdot v_{G6PDH}^{\max*}$ and $v_{PGDH}^{\max}=k_{PGDH}\cdot\mu\cdot v_{PGDH}^{\max*}$. The values of $k_{G6PDH}$ and $k_{PGDH}$ were obtained from the experimental data by assuming linear relationship between relative gene expression ($RGE$) and dilution rate or specific growth rate (Yao et al., 2011). Specifically, the following equation was assumed for gene expression of G6PDH.

| $RGE=k_{G6PDH}\cdot\mu$ | (S15c) |
| --- | --- |

The maximum velocity of G6PDH is expressed as

| $v_{G6PDH}^{\max}=k_{cat}\cdot[E]$ | (S15d) |
| --- | --- |

Divide by enzyme concentration of house-keeping gene ($[E]*$) as

| $v_{G6PDH}^{\max}/[E]*=k_{cat}\cdot[E]/[E]*$ | (S15e) |
| --- | --- |

Given that $[E]/[E]*\approx RGE$, we can derive the equation that $v_{G6PDH}^{\max}$ equals to $k_{G6PDH}\cdot\mu\cdot v_{G6PDH}^{\max*}$, where $v_{G6PDH}^{\max*}=k_{cat}\cdot[E]*$. In the same manner, $v_{PGDH}^{\max}$ of PGDH was also derived.

Non-oxidative PP pathway reactions were expressed as (Chassagnole et al., 2002)

| $v_{Rpe}=v_{Rpe}^{\max}\left([RU5P]-\frac{[X5P]}{K_{eq}}\right)$ | (S16a) |
| --- | --- |
| $v_{Rpi}=v_{Rpi}^{\max}\left([RU5P]-\frac{[R5P]}{K_{eq}}\right)$ | (S16b) |
| $v_{TktA}=v_{TktA}^{\max}\left([R5P][X5P]-\frac{[S7P][GAP/DHAP]}{K_{eq}}\right)$ | (S16c) |
| $v_{TktB}=v_{TktB}^{\max}\left([X5P][E4P]-\frac{[F6P][GAP/DHAP]}{K_{eq}}\right)$ | (S16d) |
| $v_{Tal}=v_{Tal}^{\max}\left([GAP/DHAP][S7P]-\frac{[E4P][F6P]}{K_{eq}}\right)$ | (S16e) |

**Modeling for fermentative pathways**

The lactate is formed by LDH, and the rate equation was expressed as a function of two substrates, PYR and NADH. The Hill equation was used based on the experimental observation (Dearriaga et al. 1982) as follows:

| $v_{LDH}=v_{LDH}^{\max}\cdot\frac{[NADH]^{n_{NADH}}}{K_{NADH}^{n_{NADH}}+[NADH]^{n_{NADH}}}\cdot\frac{[PYR]^{n_{PYR}}}{K_{PYR}^{n_{PYR}}+[PYR]^{n_{PYR}}}$ | (S17a) |
| --- | --- |

The formate is formed by Pfl, and its reaction was expressed as (Cintolesi et al. 2012)

| $v_{Pfl}=\frac{v_{Pfl}^{\max,f}[PYR][CoA]}{[PYR][CoA]+K_{PYR}[CoA]+K_{CoA}[PYR]}-\frac{v_{Pfl}^{\max,r}[AcCoA][FOR]}{[AcCoA][FOR]+K_{AcCoA}[FOR]+K_{FOR}[AcCoA]}$ | (S17b) |
| --- | --- |

The ethanol is formed by ADH. ALDH is used to convert AcCoA into acetaldehyde (AcAld), and the subsequent reduction of AcAld produces ethanol. These rate equations were expressed as (Hoefnagel et al. 2002)

| $v_{ALDH}=\frac{v_{ALDH}^{\max}\left([AcCoA][NADH]-\frac{[CoA][NAD][ACAL]}{K_{eq}}\right)}{\left(K_{AcCoA}K_{NADH}+\frac{K_{AcCoA}K_{NADH}[NAD]}{K_{NAD}}+K_{AcCoA}[NADH]\right)\left(1+\frac{[AcCoA]}{K_{AcCoA}}+\frac{[CoA]}{K_{CoA}}+\frac{[ACAL]}{K_{ACAL}}+\frac{[ACAL][CoA]}{K_{ACAL}K_{CoA}}\right)}$ | (S17c) |
| --- | --- |
| $v_{ADH}=\frac{v_{ADH}^{\max}\left([ACAL][NADH]-\frac{[ETH][NAD]}{K_{eq}}\right)}{\left(K_{ACAL}K_{NADH}+\frac{K_{ACAL}K_{NADH}[NAD]}{K_{NAD}}+K_{ACAL}[NADH]\right)\left(1+\frac{[ACAL]}{K_{ACAL}}+\frac{[ETH]}{K_{ETH}}\right)}$ | (S17d) |

**Modeling for acetate formation**

Acetate is formed from AcCoA by the reactions of Pta and Ack, and here we lumped these reactions together as (Kotte et al. 2010)

| $v_{PTACK}=\frac{v_{PTACK}^{\max}\frac{[AcCoA]}{K_{AcCoA}}\left(1+\frac{[AcCoA]}{K_{AcCoA}}\right)^{n-1}}{\left(1+\frac{[AcCoA]}{K_{AcCoA}}\right)^{n}+L\left(1+\frac{[PYR]}{K_{PYR}}\right)^{-n}}$ | (S18a) |
| --- | --- |

Acetate consumption rate was expressed as (Kotte et al. 2010)

| $v_{Acs}=\frac{v_{Acs}^{\max}[ACT]}{[ACT]+K_{ACT}}$ | (S18b) |
| --- | --- |

**Modeling for PDH and TCA cycle**

The equation for PDH was expressed as (Kotte et al. 2010)

| $v_{PDH}=\frac{v_{PDH}^{\max}\frac{[PYR]}{K_{PYR}}\left(1+\frac{[PYR]}{K_{PYR}}\right)^{n-1}}{\left(1+\frac{[PYR]}{K_{PYR}}\right)^{n}+L\left(1+\frac{[GOX]}{K_{GOX}}+\frac{[PYR]}{K_{I,PYR}}\right)^{n}}$ | (S19a) |
| --- | --- |

The equation for CS was expressed as (Kotte et al. 2010)

| $v_{CS}=\frac{v_{CS}^{\max}[AcCoA][OAA]}{\left(1+\frac{[\alpha KG]}{K_{\alpha KG}}\right)K_{OAA,AcCoA}K_{AcCoA}+K_{AcCoA}[OAA]+\left(1+\frac{[\alpha KG]}{K_{\alpha KG}}\right)K_{OAA}[AcCoA]+[AcCoA][OAA]}$ | (S19b) |
| --- | --- |

The rate equation for ICDH is expressed as (Kotte et al. 2010)

| $v_{ICDH}=\frac{v_{ICDH}^{\max} \frac{[CIT/ICIT]}{K_{CIT/ICIT}}\left(1+\frac{[CIT/ICIT]}{K_{CIT/ICIT}}\right)^{n-1}}{\left(1+\frac{[CIT/ICIT]}{K_{CIT/ICIT}}\right)^{n}+L\left(1+\frac{[PEP]}{K_{PEP}}\right)^{n}}$ | (S19c) |
| --- | --- |

The equation for αKGDH was expressed as (Kotte et al. 2010)

| $v_{\alpha KGDH}=\frac{v_{\alpha KGDH}^{\max}[\alpha KG]}{[\alpha KG]+K_{\alpha KG}}$ | (S19d) |
| --- | --- |

Here, the forward and backward fluxes of SDH/Frd reactions used by Usuda et al. (2010) were considered as SDH and Frd reactions, respectively.

| $v_{SDH}=\frac{v_{SDH}^{\max}[SUC^{in}]}{K_{SUC}+[SUC^{in}]+\frac{K_{SUC}[FUM/MAL]}{K_{FUM/MAL}}}$ | (S19e) |
| --- | --- |
| $v_{Frd}=\frac{v_{Frd}^{\max}[FUM/MAL]}{K_{FUM/MAL}+[FUM/MAL]+\frac{K_{FUM/MAL}[SUC^{in}]}{K_{SUC}}}$ | (S19f) |

The MDH reaction is expressed as (Usuda et al. 2010)

| $v_{MDH}=\frac{v_{MDH}^{\max,f}K_{OAA}K_{NADH}[FUM/MAL][NAD]-v_{MDH}^{\max,r}K_{FUM/MAL}K_{NAD}[OAA][NADH]}{\left(K_{FUM/MAL}K_{NAD}+K_{NAD}[FUM/MAL]+\frac{K_{FUM/MAL}K_{NAD}[OAA]}{K_{OAA}}\right)\left(K_{OAA}K_{NADH}+K_{OAA}[NADH]+\frac{K_{OAA}K_{NADH}[NAD]}{K_{NAD}}\right)}$ | (S19g) |
| --- | --- |

**Modeling for glyoxylate pathways**

The rate equations for glyoxylate pathway were expressed as (Kotte et al. 2010)

| $v_{Icl}=\frac{v_{Icl}^{\max} \frac{[CIT/ICIT]}{K_{CIT/ICIT}}\left(1+\frac{[CIT/ICIT]}{K_{CIT/ICIT}}\right)^{n-1}}{\left(1+\frac{[CIT/ICIT]}{K_{CIT/ICIT}}\right)^{n}+L\left(1+\frac{[PEP]}{K_{PEP}}+\frac{[GAP/\sim/2PG]}{K_{GAP/\sim/2PG}}+\frac{[\alpha KG]}{K_{\alpha KG}}\right)^{n}}$ | (S20a) |
| --- | --- |
| $v_{MS}=\frac{v_{MS}^{\max} [GOX][AcCoA]}{K_{GOXAcCoA}K_{AcCoA}+K_{AcCoA}[GOX]+K_{GOX}[AcCoA]+[GOX][AcCoA]}$ | (S20b) |

**Modeling for gluconeogenesis**

The equation for Fbp, Pps, Pck, and Mez were expressed as (Kotte et al. 2010)

| $v_{Fbp}=\frac{v_{Fbp}^{\max} \frac{[FBP]}{K_{FBP}}\left(1+\frac{[FBP]}{K_{FBP}}\right)^{n-1}}{\left(1+\frac{[FBP]}{K_{FBP}}\right)^{n}+L\left(1+\frac{[PEP]}{K_{PEP}}\right)^{-n}}$ | (S21a) |
| --- | --- |
| $v_{Pps}=\frac{v_{Pps}^{\max} \frac{[PYR]}{K_{PYR}}\left(1+\frac{[PYR]}{K_{PYR}}\right)^{n-1}}{\left(1+\frac{[PYR]}{K_{PYR}}\right)^{n}+L\left(1+\frac{[PEP]}{K_{PEP}}\right)^{n}}$ | (S21b) |
| $v_{Pck}=\frac{v_{Pck}^{\max}[OAA]}{[OAA]+K_{OAA}\left(1+\frac{[PEP]}{K_{PEP}}\right)}$ | (S21c) |
| $v_{Mez}=\frac{v_{Mez}^{\max}\frac{[FUM/MAL]}{K_{FUM/MAL}}\left(1+\frac{[FUM/MAL]}{K_{FUM/MAL}}\right)^{n-1}}{\left(1+\frac{[FUM/MAL]}{K_{FUM/MAL}}\right)^{n}+L\left(1+\frac{[AcCoA]}{K_{AcCoA}}+\frac{[cAMP]}{K_{cAMP}}\right)^{n}}$ | (S21d) |

**Modeling for anaplerotic reaction**

The rate equation for Ppc was expressed as (Kotte et al. 2010)

| $v_{Ppc}=\frac{v_{Ppc}^{\max}\frac{[PEP]}{K_{PEP}}\left(1+\frac{[PEP]}{K_{PEP}}\right)^{n-1}}{\left(1+\frac{[PEP]}{K_{PEP}}\right)^{n}+L\left(1+\frac{[FBP]}{K_{FBP}}\right)^{-n}}$ | (S22) |
| --- | --- |

**Modeling for respiratory chain**

The rate reactions of Nuo and Ndh were expressed as (Henkel et al. 2014)

| $v_{Nuo}=v_{Nuo}^{\max}\cdot\frac{[Q]}{K_{m,Nuo,Q}+\left[Q\right]}\cdot\frac{[NADH]}{K_{m,Nuo,NADH}+\left[NADH\right]}$ | (S23a) |
| --- | --- |
| $v_{Ndh}=v_{Ndh}^{\max}\cdot\frac{[Q]}{K_{m,Ndh,Q}+\left[Q\right]}\cdot\frac{[NADH]}{K_{m,Ndh,NADH}+\left[NADH\right]}$ | (S23b) |

The rate reactions of Cyo and Cyd were expressed as (Henkel et al. 2014)

| $v_{Cyo}=v_{Cyo}^{\max}\cdot\frac{[O_{2}]}{K_{m,Cyo,O_{2}}+\left[O_{2}\right]}\cdot\frac{[QH_{2}]}{K_{m,Cyo,QH_{2}}+\left[QH_{2}\right]}$ | (S23c) |
| --- | --- |
| $v_{Cyd}=v_{Cyd}^{\max}\cdot\frac{[O_{2}]}{K_{m,Cyd,O_{2}}+\left[O_{2}\right]}\cdot\frac{[QH_{2}]}{K_{m,Cyd,QH_{2}}+\left[QH_{2}\right]}$ | (S23d) |

Assuming that the oxygen level available to the cell (or in the cytoplasm) is lower than the dissolved oxygen (DO) level in the culture medium (Potzkei et al., 2012), we introduced the model parameter $k_{O_{2}}$, where DO concentration in the culture medium multiplied by $k_{O_{2}}$is $[O_{2}]$, such that $[O_{2}]=k_{O_{2}}[DO_{2}]$.

The quinol synthetic rate was expressed based on Henkel et al. (2014)

| $v_{Syn,QH_{2}}=\mu\cdot p_{syn,QH_{2}}$ | (S23e) |
| --- | --- |

**Modeling for SUC transport**

SUC transport was expressed as

| $v_{SUC_{trans}}=\frac{v_{SUC_{trans}}^{\max}[SUC^{in}]}{K_{SUC}+[SUC^{in}]}$ | (S24) |
| --- | --- |

**Modeling for biosynthetic pathway**

The biosynthetic fluxes were expressed as growth dependent as follows:

| $v_{Bio,G6P/F6P}=\left(k_{Bio,G6P/F6P}^{+}\cdot\mu+k_{Bio,G6P/F6P}^{++}\right)[G6P/F6P]$ | (S25a) |
| --- | --- |
| $v_{Bio,GAP/DHAP}=\left(k_{Bio,GAP/DHAP}^{+}\cdot\mu+k_{Bio,GAP/DHAP}^{++}\right)[GAP/DHAP]$ | (S25b) |
| $v_{Bio,PEP}=\left(k_{Bio,PEP}^{+}\cdot\mu+k_{Bio,PEP}^{++}\right)[PEP]$ | (S25c) |
| $v_{Bio,PYR}=\left(k_{Bio,PYR}^{+}\cdot\mu+k_{Bio,PYR}^{++}\right)[PYR]$ | (S25d) |
| $v_{Bio,AcCoA}=\left(k_{Bio,AcCoA}^{+}\cdot\mu+k_{Bio,AcCoA}^{++}\right)[AcCoA]$ | (S25e) |
| $v_{Bio,R5P}=\left(k_{Bio,R5P}^{+}\cdot\mu+k_{Bio,R5P}^{++}\right)[R5P]$ | (S25f) |
| $v_{Bio,E4P}=\left(k_{Bio,E4P}^{+}\cdot\mu+k_{Bio,E4P}^{++}\right)[E4P]$ | (S25g) |
| $v_{Bio,\alpha KG}=\left(k_{Bio,\alpha KG}^{+}\cdot\mu+k_{Bio,\alpha KG}^{++}\right)[\alpha KG]$ | (S25h) |
| $v_{Bio,OAA}=\left(k_{Bio,OAA}^{+}\cdot\mu+k_{Bio,OAA}^{++}\right)[OAA]$ | (S25i) |

**Modeling for transcription factors**

For the interactions of the transcription factors, following equations were used (Kotte et al., 2010)

| $v_{cAMP-Crp}=p_{cAMP-Crp}\left\{\frac{\left([Crp]+[cAMP-Crp]\right)[cAMP]^{n}}{[cAMP]^{n}+K_{cAMP}^{n}}-[cAMP-Crp]\right\}$ | (S26a) |
| --- | --- |
| $v_{Cra-FBP}=p_{Cra-FBP}\left\{\frac{\left([Cra]+[Cra-FBP]\right)[FBP]^{n}}{[FBP]^{n}+K_{FBP}^{n}}-[Cra-FBP]\right\}$ | (S26b) |
| $v_{PdhR-PYR}=p_{PdhR-PYR}\left\{\frac{\left([PdhR]+[PdhR-PYR]\right)[PYR]^{n}}{[PYR]^{n}+K_{PYR}^{n}}-[PdhR-PYR]\right\}$ | (S26c) |

**Modeling for extracellular metabolite uptake/excretion rates**

The extracellular metabolite uptake/excretion rates were expressed by the following equations:

| $v_{GLC,uptake}=M_{w}^{GLC}\cdot p_{uc}\cdot[X]\cdot\left(v_{PTS4}+v_{NPTS}\right)$ | (S27a) |
| --- | --- |
| $v_{ACT,excrete}=M_{w}^{ACT}\cdot p_{uc}\cdot[X]\cdot v_{PTACK}$ | (S27b) |
| $v_{ACT,uptake}=M_{w}^{ACT}\cdot p_{uc}\cdot[X]\cdot v_{Acs}$ | (S27c) |
| $v_{LAC,excrete}=M_{w}^{LAC}\cdot p_{uc}\cdot[X]\cdot v_{LDH}$ | (S27d) |
| $v_{FOR,excrete}=M_{w}^{FOR}\cdot p_{uc}\cdot[X]\cdot v_{Pfl}$ | (S27e) |
| $v_{ETH,excrete}=M_{w}^{ETH}\cdot p_{uc}\cdot[X]\cdot v_{ADH}$ | (S27f) |

**Effects of transcription factors on main metabolic pathways**

As explained in the main text, a set of metabolic pathway genes are under control of the transcription factors. In the present model, such effects of the transcription factors reflect the metabolic pathway fluxes (Fig. 1 in the main text). Specifically, the maximum velocity ($v_{\bullet}^{\max}$) of the enzymatic reaction may be modified as a function of the transcription factors as follows:

| $v_{\bullet}^{\max}=v_{\bullet}^{\max'}\cdot\left(1+\sum_{i} TF_{i}\right)\cdot\prod_{j} \left(1-TF_{j}\right)$ | (S28a) |
| --- | --- |

where $TF_{i}$ or $TF_{j}$ are the activities of the transcription factor (Hardiman et al., 2010). The maximum velocities of the Pfl and Frd reactions may be expressed as follows because those reactions are not active under aerobic cultivation, but induced as the oxygen level decreases:

| $v_{\bullet}^{\max}=v_{\bullet}^{\max'}\cdot\sum_{i} TF_{i}\cdot\prod_{j} \left(1-TF_{j}\right)$ | (S28b) |
| --- | --- |

The activities of the transcription factor such as cAMP-Crp, Cra, and PdhR may be expressed as follows:

| $TF_{cAMP-Crp}=\frac{[cAMP-Crp]}{[cAMP-Crp]+K_{cAMP-Crp}}$ | (S29a) |
| --- | --- |
| $TF_{Cra}=\frac{[Cra]}{[Cra]+K_{Cra}}$ | (S29b) |
| $TF_{PdhR}=\frac{[PdhR]}{[PdhR]+K_{PdhR}}$ | (S29c) |

The activity of the IclR may be expressed as a function of the cAMP-Crp as follows:

| $TF_{IclR}=\frac{[cAMP-Crp]^{n}}{[cAMP-Crp]^{n}+K_{IclR}^{n}}$ | (S29d) |
| --- | --- |

where *crp* knockout mutant showed the higher *iclR* gene expression than wild type (Yao et al., 2011).

**References**

Bettenbrock K, Fischer S, Kremling A, Jahreis K, Sauter T, Gilles ED: A quantitative approach to catabolite repression in *Escherichia coli*. J Biol Chem 2006, **281**:2578-2584.

Chassagnole C, Noisommitt-Rizzi N, Schmid JW, Mauch K, Reuss M: Dynamic modeling of the central carbon metabolism of *Escherichia coli*. Biotechnol Bioeng 2002, **79**:53–73.

Dearriaga D, Soler J, Cadenas E: Influence of ph on the allosteric properties of lactate-dehydrogenase activity of *Phycomyces blakesleeanus*. Biochem J 1982, **203**:393-400.

Hardiman T, Meinhold H, Hofmann J, Ewald JC, Siemann-Herzberg M, Reuss M: Prediction of kinetic parameters from DNA-binding site sequences for modeling global transcription dynamics in *Escherichia coli*. Metab Eng 2010, **12**:196-211.

Henkel SG, Ter Beek A, Steinsiek S, Stagge S, Bettenbrock K, de Mattos MJT, Sauter T, Sawodny O, Ederer M: Basic regulatory principles of *Escherichia coli*'s electron transport chain for varying oxygen conditions. PLoS ONE 2014, **9**:e107640.

Hoefnagel MHN, Starrenburg MJC, Martens DE, Hugenholtz J, Kleerebezem M, Van Swam II, Bongers R, Westerhoff HV, Snoep JL: Metabolic engineering of lactic acid bacteria, the combined approach: kinetic modelling, metabolic control and experimental analysis. Microbiology 2002, **148**:1003-1013.

Kotte O, Zaugg JB, Heinemann M: Bacterial adaptation through distributed sensing of metabolic fluxes. Mol Syst Biol 2010, **6**:355.

Kremling A, Bettenbrock K, Laube B, Jahreis K, Lengeler JW, Gilles ED: The organization of metabolic reaction networks. III. Application for diauxic growth on glucose and lactose. Metab Eng 2001, **3**:362-379.

Potzkei J, Kunze M, Drepper T, Gensch T, Jaeger KE, Büchs J: Real-time determination of intracellular oxygen in bacteria using a genetically encoded FRET-based biosensor. BMC Biol 2012, **10**:28.

Usuda Y, Nishio Y, Iwatani S, Van Dien SJ, Imaizumi A, Shimbo K, Kageyama N, Iwahata D, Miyano H, Matsui K: Dynamic modeling of *Escherichia coli* metabolic and regulatory systems for amino-acid production. J Biotechnol 2010, **147**:17-30.

Wolf RE Jr, Prather DM, Shea FM: Growth-rate-dependent alteration of 6-phosphogluconate dehydrogenase and glucose 6-phosphate dehydrogenase levels in *Escherichia coli* K-12. J Bacteriol 1979, **139**:1093-1096.

Yao R, Hirose Y, Sarkar D, Nakahigashi K, Ye Q, Shimizu K: Catabolic regulation analysis of *Escherichia coli* and its *crp*, *mlc*, *mgsA*, *pgi* and *ptsG* mutants. Microb Cell Fact 2011, **10**:67.
