# Supplementary material for: Modeling and simulation of the redox regulation of the metabolism in Escherichia coli at different oxygen concentrations
Source: Biotechnol Biofuels. 2017 Jul 14;10:183. doi: 10.1186/s13068-017-0867-0 (PMC5512849; doi:10.1186/s13068-017-0867-0)
Supplement: Supplementary file 2 — Additional file 2. Model parameters. [file 13068_2017_867_MOESM2_ESM.doc]

**Additional file 2**

**Model parameters**

Table S1 and Table S2 show the kinetic parameters and the total concentrations for transcription factor and cofactor concentrations, respectively. Other parameters are also shown in Table S3.

**Table S1. Comprehensive list of the kinetic parameters.**

| Reaction name | Parameter | Value | Reference |
| --- | --- | --- | --- |
| PTS1 | $k_{1}$ | 116 [gDCW/gProt.s] | Kotte et al. 2010 |
|  | $k_{-1}$ | 46.3 [gDCW/gProt.s] | Kotte et al. 2010 |
| PTS4 | $v_{PTS4}^{\max'}$ | 4.38 [μmol/gDCW.s] | *1 |
|  | $K_{EIIA\sim P}$ | 8.5E-3 [gProt/gDCW] | Kotte et al. 2010 |
|  | $K_{Glc}$ | 1.2E-3 [g/l] | Kotte et al. 2010 |
| NPTS | $v_{NPTS}^{\max'}$ | 0.167 [μmol/gDCW.s] | *1 |
|  | $K_{S}$ | 1.0E-2 [μmol/gDCW] | Bettenbrock et al. 2006 |
|  | $K_{I}$ | 5.0E-6 [μmol/gDCW] | Bettenbrock et al. 2006 |
| Glk | $v_{Glk}^{\max'}$ | 5.556 [μmol/gDCW.s] | *1 |
|  | $K_{S}$ | 16.0 [μmol/gDCW] | Bettenbrock et al. 2006 |
|  | $K_{I}$ | 0.1 [μmol/gDCW] | Bettenbrock et al. 2006 |
| Pfk | $v_{Pfk}^{\max'}$ | 92.511 [μmol/gDCW.s] | *1 |
|  | $K_{G6P/F6P}$ | 2.2E-2 [μmol/gDCW] | Kotte et al. 2010 |
|  | $K_{PEP}$ | 0.138 [μmol/gDCW] | Kotte et al. 2010 |
|  | $L$ | 9.5E+7 | Kotte et al. 2010 |
|  | $n$ | 4 | Kotte et al. 2010 |
| Fba | $v_{Fba}^{\max',f}$ | 10.195 [μmol/gDCW.s] | *1 |
|  | $v_{Fba}^{\max',r}$ | 10.195 [μmol/gDCW.s] | *1 |
|  | $K_{FBP}$ | 5.92 [μmol/gDCW] | Kotte et al. 2010 |
|  | $K_{GAP/DHAP}$ | 16.6 [μmol/gDCW] | Kotte et al. 2010 |
| L_Emp | $v_{L\_Emp}^{\max',f}$ | 10.195 [μmol/gDCW.s] | *1 |
|  | $v_{L\_Emp}^{\max',r}$ | 10.195 [μmol/gDCW.s] | *1 |
|  | $K_{GAP/DHAP}$ | 4.76 [μmol/gDCW] | Kotte et al. 2010 |
|  | $K_{PEP}$ | 1.11 [μmol/gDCW] | Kotte et al. 2010 |
| Pyk | $v_{Pyk}^{\max'}$ | 49.502 [μmol/gDCW.s] | *1 |
|  | $K_{PEP}$ | 5.0 [μmol/gDCW] | Kotte et al. 2010 |
|  | $K_{FBP}$ | 0.413 [μmol/gDCW] | Kotte et al. 2010 |
|  | $L$ | 1.0E+5 | Kotte et al. 2010 |
|  | $n$ | 4 | Kotte et al. 2010 |
| G6PDH | $v_{G6PDH}^{\max*}$ | 2.447 [μmol/gDCW.s] | *1 |
|  | $k_{G6PDH}$ | 1.220E+4 [s] | Yao et al., 2011 |
|  | $K_{G6P}$ | 25.532 [μmol/gDCW] ^*2^ | Chassagnole et al. 2002 |
|  | $K_{NADP}$ | 0.044 [μmol/gDCW] ^*2^ | Chassagnole et al. 2002 |
|  | $K_{NADPH\_G6Pinh}$ | 11.401 [μmol/gDCW] ^*2^ | Chassagnole et al. 2002 |
|  | $K_{NADPH\_NADPinh}$ | 0.018 [μmol/gDCW] ^*2^ | Chassagnole et al. 2002 |
| PGDH | $v_{PGDH}^{\max*}$ | 28.781 [μmol/gDCW.s] | *1 |
|  | $k_{PGDH}$ | 1.004E+4 [s] | Yao et al., 2011 |
|  | $K_{6PG}$ | 66.489 [μmol/gDCW] ^*2^ | Chassagnole et al. 2002 |
|  | $K_{NADP}$ | 0.090 [μmol/gDCW] ^*2^ | Chassagnole et al. 2002 |
|  | $K_{NADPHinh}$ | 0.025 [μmol/gDCW] ^*2^ | Chassagnole et al. 2002 |
|  | $K_{ATPinh}$ | 368.794 [μmol/gDCW] ^*2^ | Chassagnole et al. 2002 |
| Rpe | $v_{Rpe}^{\max'}$ | 11.949 [μmol/gDCW.s] ^*2^ | Chassagnole et al. 2002 |
|  | $K_{eq}$ | 1.4 | Chassagnole et al. 2002 |
| Rpi | $v_{Rpi}^{\max'}$ | 8.579 [μmol/gDCW.s] ^*2^ | Chassagnole et al. 2002 |
|  | $K_{eq}$ | 4.0 | Chassagnole et al. 2002 |
| TktA | $v_{TktA}^{\max'}$ | 16.797 [μmol/gDCW.s] ^*2^ | Chassagnole et al. 2002 |
|  | $K_{eq}$ | 1.2 | Chassagnole et al. 2002 |
| TktB | $v_{TktB}^{\max'}$ | 153.473 [μmol/gDCW.s] ^*2^ | Chassagnole et al. 2002 |
|  | $K_{eq}$ | 10.0 | Chassagnole et al. 2002 |
| Tal | $v_{Tal}^{\max'}$ | 19.276 [μmol/gDCW.s] ^*2^ | Chassagnole et al. 2002 |
|  | $K_{eq}$ | 1.05 | Chassagnole et al. 2002 |
| LDH | $v_{LDH}^{\max'}$ | 51.7 [μmol/gDCW.s] | *1 |
|  | $K_{NADH}$ | 0.9 [μmol/gDCW] | *1 |
|  | $K_{PYR}$ | 26.7 [μmol/gDCW] | *1 |
|  | $n_{NADH}$ | 3 | *1 |
|  | $n_{PYR}$ | 2 | *1 |
| Pfl | $v_{Pfl}^{\max',f}$ | 14.7 [μmol/gDCW.s] | *1 |
|  | $v_{Pfl}^{\max',r}$ | 3.4 [μmol/gDCW.s] | *1 |
|  | $K_{PYR}$ | 4.5 [μmol/gDCW] | *1 |
|  | $K_{CoA}$ | 0.782 [μmol/gDCW] ^*3^ | Cintolesi et al. 2012 |
|  | $K_{AcCoA}$ | 5.862 [μmol/gDCW] ^*3^ | Cintolesi et al. 2012 |
|  | $K_{FOR}$ | 1.128 [g/l] ^*3^ | Cintolesi et al. 2012 |
| ALDH | $v_{ALDH}^{\max'}$ | 63.9 [μmol/gDCW.s] | *1 |
|  | $K_{AcCoA}$ | 0.012 [μmol/gDCW] ^*2^ | Hoefnagel et al. 2002 |
|  | $K_{NADH}$ | 0.443 [μmol/gDCW] | *1 |
|  | $K_{NAD}$ | 0.142 [μmol/gDCW] ^*2^ | Hoefnagel et al. 2002 |
|  | $K_{CoA}$ | 0.014 [μmol/gDCW] ^*2^ | Hoefnagel et al. 2002 |
|  | $K_{ACAL}$ | 17.731 [μmol/gDCW] ^*2^ | Hoefnagel et al. 2002 |
|  | $K_{eq}$ | 27.73 [μmol/gDCW] | *1 |
| ADH | $v_{ADH}^{\max'}$ | 57.97 [μmol/gDCW.s] | *1 |
|  | $K_{ACAL}$ | 0.053 [μmol/gDCW] ^*2^ | Hoefnagel et al. 2002 |
|  | $K_{NADH}$ | 2.1 [μmol/gDCW] | *1 |
|  | $K_{NAD}$ | 0.142 [μmol/gDCW] ^*2^ | Hoefnagel et al. 2002 |
|  | $K_{ETH}$ | 7.107E-04 [g/l] ^*2, 3^ | Hoefnagel et al. 2002 |
|  | $K_{eq}$ | 2.191E+04 [μmol/gDCW] ^*2^ | Hoefnagel et al. 2002 |
| PTACK | $v_{PTACK}^{\max'}$ | 2.2 [μmol/gDCW.s] | *1 |
|  | $K_{AcCoA}$ | 2.2E-2 [μmol/gDCW] | Kotte et al. 2010 |
|  | $K_{PYR}$ | 2.2E-2 [μmol/gDCW] | Kotte et al. 2010 |
|  | $L$ | 6.39E+5 | Kotte et al. 2010 |
|  | $n$ | 2 | Kotte et al. 2010 |
| Acs | $v_{Acs}^{\max'}$ | 0.729 [μmol/gDCW.s] | *1 |
|  | $K_{ACT}$ | 1.0E-3 [g/l] | Kotte et al. 2010 |
| PDH | $v_{PDH}^{\max'}$ | 5.667 [μmol/gDCW.s] | *1 |
|  | $K_{PYR}$ | 0.128 [μmol/gDCW] | Kotte et al. 2010 |
|  | $K_{GOX}$ | 0.218 [μmol/gDCW] | Kotte et al. 2010 |
|  | $K_{I,PYR}$ | 0.231 [μmol/gDCW] | Kotte et al. 2010 |
|  | $L$ | 3.4 | Kotte et al. 2010 |
|  | $n$ | 2.65 | Kotte et al. 2010 |
| CS | $v_{CS}^{\max'}$ | 2.115 [μmol/gDCW.s] | *1 |
|  | $K_{AcCoA}$ | 0.212 [μmol/gDCW] | Kotte et al. 2010 |
|  | $K_{OAA}$ | 0.029 [μmol/gDCW] | Kotte et al. 2010 |
|  | $K_{OAA,AcCoA}$ | 0.029 [μmol/gDCW] | Kotte et al. 2010 |
|  | $K_{\alpha KG}$ | 0.63 [μmol/gDCW] | Kotte et al., 2010 |
| ICDH | $v_{ICDH}^{\max'}$ | 7.887 [μmol/gDCW.s] | *1 |
|  | $K_{CIT/ICIT}$ | 1.6E-4 [μmol/gDCW] | Kotte et al. 2010 |
|  | $K_{PEP}$ | 0.334 [μmol/gDCW] | Kotte et al. 2010 |
|  | $L$ | 127.0 | Kotte et al. 2010 |
|  | $n$ | 2 | Kotte et al. 2010 |
| αKGDH | $v_{\alpha KGDH}^{\max'}$ | 1.183 [μmol/gDCW.s] | *1 |
|  | $K_{\alpha KG}$ | 0.548 [μmol/gDCW] | Kotte et al. 2010 |
| SDH | $v_{SDH}^{\max'}$ | 1.810 [μmol/gDCW.s] | *1 |
|  | $K_{SUC}$ | 0.230 [μmol/gDCW] ^*3^ | Usuda et al. 2010 |
|  | $K_{FUM/MAL}$ | 0.575 [μmol/gDCW] ^*3^ | Usuda et al. 2010 |
| Frd | $v_{Frd}^{\max'}$ | 2.5 [μmol/gDCW.s] | *1 |
|  | $K_{FUM/MAL}$ | 0.575 [μmol/gDCW] ^*3^ | Usuda et al. 2010 |
|  | $K_{SUC}$ | 0.230 [μmol/gDCW] ^*3^ | Usuda et al. 2010 |
| MDH | $v_{MDH}^{\max',f}$ | 12.677 [μmol/gDCW.s] | *1 |
|  | $v_{MDH}^{\max',r}$ | 54.332 [μmol/gDCW.s] | *1 |
|  | $K_{FUM/MAL}$ | 29.8 [μmol/gDCW] | *1 |
|  | $K_{NAD}$ | 29.885 [μmol/gDCW] ^*3^ | Usuda et al. 2010 |
|  | $K_{OAA}$ | 0.563 [μmol/gDCW] | *1 |
|  | $K_{NADH}$ | 7.012 [μmol/gDCW] ^*3^ | Usuda et al. 2010 |
| Ppc | $v_{Ppc}^{\max'}$ | 1.0 [μmol/gDCW.s] | *1 |
|  | $K_{PEP}$ | 0.048 [μmol/gDCW] | Kotte et al. 2010 |
|  | $K_{FBP}$ | 0.408 [μmol/gDCW] | Kotte et al. 2010 |
|  | $L$ | 5.2E+6 | Kotte et al. 2010 |
|  | $n$ | 3 | Kotte et al. 2010 |
| Icl | $v_{Icl}^{\max'}$ | 1.232 [μmol/gDCW.s] | *1 |
|  | $K_{CIT/ICIT}$ | 0.0022 [μmol/gDCW] | *1 |
|  | $K_{PEP}$ | 0.055 [μmol/gDCW] | Kotte et al. 2010 |
|  | $K_{GAP/\sim/2PG}$ | 0.72 [μmol/gDCW] | Kotte et al. 2010 |
|  | $K_{\alpha KG}$ | 0.827 [μmol/gDCW] | Kotte et al. 2010 |
|  | $L$ | 5.01 | *1 |
|  | $n$ | 4 | Kotte et al. 2010 |
| MS | $v_{MS}^{\max'}$ | 2.144 [μmol/gDCW.s] | *1 |
|  | $K_{GOX}$ | 0.95 [μmol/gDCW] | Kotte et al. 2010 |
|  | $K_{AcCoA}$ | 0.755 [μmol/gDCW] | Kotte et al. 2010 |
|  | $K_{GOXAcCoA}$ | 0.719 [μmol/gDCW] | Kotte et al. 2010 |
| Fbp | $v_{Fbp}^{\max'}$ | 0.114 [μmol/gDCW.s] | *1 |
|  | $K_{FBP}$ | 3.0E-3 [μmol/gDCW] | Kotte et al. 2010 |
|  | $K_{PEP}$ | 0.3 [μmol/gDCW] | Kotte et al. 2010 |
|  | $L$ | 4.0E+6 | Kotte et al. 2010 |
|  | $n$ | 4 | Kotte et al. 2010 |
| Pps | $v_{Pps}^{\max'}$ | 4.1E-3 [μmol/gDCW.s] | *1 |
|  | $K_{PYR}$ | 1.77E-3 [μmol/gDCW] | Kotte et al. 2010 |
|  | $K_{PEP}$ | 1.0E-3 [μmol/gDCW] | Kotte et al. 2010 |
|  | $L$ | 1.0E-79 | Kotte et al. 2010 |
|  | $n$ | 2 | Kotte et al. 2010 |
| Pck | $v_{Pck}^{\max'}$ | 0.087 [μmol/gDCW.s] | *1 |
|  | $K_{OAA}$ | 0.184 [μmol/gDCW] | Kotte et al. 2010 |
|  | $K_{PEP}$ | 1000 [μmol/gDCW] | Kotte et al. 2010 |
| Mez | $v_{Mez}^{\max'}$ | 0.707 [μmol/gDCW.s] | *1 |
|  | $K_{FUM/MAL}$ | 6.24E-3 [μmol/gDCW] | Kotte et al. 2010 |
|  | $K_{AcCoA}$ | 3.64 [μmol/gDCW] | Kotte et al. 2010 |
|  | $K_{cAMP}$ | 6.54 [μmol/gDCW] | Kotte et al. 2010 |
|  | $L$ | 1.04E+5 | Kotte et al. 2010 |
|  | $n$ | 1.33 | Kotte et al. 2010 |
| SUC_trans_ | $v_{SUC_{trans}}^{\max'}$ | 0.5 [μmol/gDCW.s] | *1 |
|  | $K_{SUC}$ | 16.0 [μmol/gDCW] | *1 |
| Cya | $v_{Cya}^{\max'}$ | 0.993 [μmol/gDCW.s] | Kotte et al. 2010 |
|  | $K_{EIIA\sim P}$ | 1.7E-3 [gProt/gDCW] | Kotte et al. 2010 |
| cAMPdegr | $v_{Degr}^{\max'}$ | 1.0 [μmol/gDCW.s] | *1 |
|  | $K_{cAMP}$ | 0.1 [μmol/gDCW] | Kotte et al. 2010 |
| cAMP-Crp  association/  dissociation | $p_{cAMP-Crp}$ | 1.0E+8 [/s] | Kotte et al. 2010 |
|  | $K_{cAMP}$ | 0.895 [μmol/gDCW] | Kotte et al. 2010 |
|  | $n$ | 1 | Kotte et al. 2010 |
| Cra-FBP  association/  dissociation | $p_{Cra-FBP}$ | 100 [/s] | Kotte et al. 2010 |
|  | $K_{FBP}$ | 1.36 [μmol/gDCW] | Kotte et al. 2010 |
|  | $n$ | 2 | Kotte et al. 2010 |
| PdhR-PYR  association/  dissociation | $p_{PdhR-PYR}$ | 100 [/s] | Kotte et al. 2010 |
|  | $K_{PYR}$ | 0.164 [μmol/gDCW] | Kotte et al. 2010 |
|  | $n$ | 1 | Kotte et al. 2010 |
| Nuo | $v_{Nuo}^{\max'}$ | 708.333 [μmol/gDCW.s] | *1 |
|  | $K_{m,Nuo,Q}$ | 0.34 [μmol/gDCW] | Henkel et al. 2014 |
|  | $K_{m,Nuo,NADH}$ | 20 [μmol/gDCW] | Henkel et al. 2014 |
| Ndh | $v_{Ndh}^{\max'}$ | 11.111 [μmol/gDCW.s] | *1 |
|  | $K_{m,Ndh,Q}$ | 0.34 [μmol/gDCW] | Henkel et al. 2014 |
|  | $K_{m,Ndh,NADH}$ | 20 [μmol/gDCW] | Henkel et al. 2014 |
| Cyo | $v_{Cyo}^{\max'}$ | 800.0 [μmol/gDCW.s] | *1 |
|  | $K_{m,Cyo,O_{2}}$ | 2E-4 [mM] | Alexeeva et al. 2002 |
|  | $K_{m,Cyo,QH_{2}}$ | 25.0 [μmol/gDCW] | *1 |
| Cyd | $v_{Cyd}^{\max'}$ | 31.558 [μmol/gDCW.s] | *1 |
|  | $K_{m,Cyd,O_{2}}$ | 2.4E-5 [mM] | Alexeeva et al. 2002 |
|  | $K_{m,Cyd,QH_{2}}$ | 10.0 [μmol/gDCW] | *1 |
| QH_2_  synthesis | $p_{syn,QH_{2}}$ | 2.16 [μmol/gDCW] | *1 |
| G6P/F6P  synthesis | $k_{Bio,G6P/F6P}^{+}$ | 554.4 [-] | *1 |
|  | $k_{Bio,G6P/F6P}^{++}$ | 0.002 [/s] | *1 |
| GAP/DHAP  synthesis | $k_{Bio,GAP/DHAP}^{+}$ | 352.8 [-] | *1 |
|  | $k_{Bio,GAP/DHAP}^{++}$ | 0.002 [/s] | *1 |
| PEP  synthesis | $k_{Bio,PEP}^{+}$ | 3.046E+3 | *1 |
|  | $k_{Bio,PEP}^{++}$ | 0.002 [/s] | *1 |
| PYR  synthesis | $k_{Bio,PYR}^{+}$ | 39.816 [-] | *1 |
|  | $k_{Bio,PYR}^{++}$ | 0.002 [/s] | *1 |
| AcCoA  synthesis | $k_{Bio,AcCoA}^{+}$ | 135.36 [-] | *1 |
|  | $k_{Bio,AcCoA}^{++}$ | 0.035 [/s] | *1 |
| R5P  synthesis | $k_{Bio,R5P}^{+}$ | 72.0 [-] | *1 |
|  | $k_{Bio,R5P}^{++}$ | 0.002 [/s] | *1 |
| E4P  synthesis | $k_{Bio,E4P}^{+}$ | 72.0 [-] | *1 |
|  | $k_{Bio,E4P}^{++}$ | 0.002 [/s] | *1 |
| αKG  synthesis | $k_{Bio,\alpha KG}^{+}$ | 7.042E+3 [-] | *1 |
|  | $k_{Bio,\alpha KG}^{++}$ | 0.002 [/s] | *1 |
| OAA  synthesis | $k_{Bio,OAA}^{+}$ | 46.080 [-] | *1 |
|  | $k_{Bio,OAA}^{++}$ | 0.002 [/s] | *1 |
| cAMP-Crp | $K_{cAMP-Crp}$ | 1.2E-3 [gProt/gDCW] | *1 |
| Cra | $K_{Cra}$ | 1.5E-2 [gProt/gDCW] | *1 |
| PdhR | $K_{PdhR}$ | 1.0E-3 [gProt/gDCW] | *1 |
| IclR | $K_{IclR}$ | 0.003 [gProt/gDCW] | *1 |
|  | $n$ | -3 | *1 |
| ArcA | $K_{ArcA}$ | 0.5 [μmol/gDCW] | *1 |
|  | $n$ | -5 | *1 |
| Fnr | $K_{Fnr}$ | 5.0E-6 [mM] | *1 |
|  | $n$ | -5 | *1 |

^*1^ The parameter values were tuned in the present model.

^*2^ The values were converted into [μmol/gDCW] using cell density 564 [gDCW/L cell volume] (Chassagnole et al. 2002).

^*3^ The values were converted into [μmol/gDCW], where the cell concentration was assumed to be a certain value (Chassagnole et al. 2002).

**Table S2. Total concentrations for transcription factor and cofactor concentrations.**

| Concentration | Value | Reference |
| --- | --- | --- |
| $[Crp]_{total}$ | 7.29E-3 [gProt/gDCW] | Kotte et al. 2010 |
| $[Cra]_{total}$ | 7.29E-3 [gProt/gDCW] | Kotte et al. 2010 |
| $[PdhR]_{total}$ | 7.29E-3 [gProt/gDCW] | Kotte et al. 2010 |
| $[IclR]$ | 7.29E-3 [gProt/gDCW] | Kotte et al. 2010 |
| $[ATP]$ | 7.571 [μmol/gDCW] ^*1^ | Chassagnole et al. 2002 |
| $[NADP]$ | 0.346 [μmol/gDCW] ^*1^ | Chassagnole et al. 2002 |
| $[NADPH]$ | 0.110 [μmol/gDCW] ^*1^ | Chassagnole et al. 2002 |
| $[NA*]_{total}$ | 12.90 [μmol/gDCW] | Berrios-Rivera et al. 2002 |
| $[CoA]$ | 0.887 [μmol/gDCW] | assumed |

^*1^ The values were converted into [μmol/gDCW] using cell density 564 [gDCW/L cell volume] (Chassagnole et al. 2002).

**Table S3. Other parameters.**

|  | Value | Reference |
| --- | --- | --- |
| $k_{ATP}$ | 1E-5 [gDCW/μmol] | Matsuoka and Shimizu 2013 |
| $k_{O_{2}}$ | 0.001 | assumed |
| $\left[DO_{2}\right]*$ | 0.214 [mM] | - |
| $M_{w}^{GLC}$ | 180.156 | - |
| $M_{w}^{ACT}$ | 60.050 | - |
| $M_{w}^{LAC}$ | 90.08 | - |
| $M_{w}^{FOR}$ | 46.025 | - |
| $M_{w}^{ETH}$ | 46.07 | - |
| $p_{uc}$ | 9.5E-7 [gDCW/μ(OD).l] | Kotte et al. 2010 |

**References**

Alexeeva S, Hellingwerf KJ, Teixeira de Mattos MJ: Quantitative assessment of oxygen availability: Perceived aerobiosis and its effect on flux distribution in the respiratory chain of *Escherichia coli*. J Bacteriol 2002, **184**:1402-1406.

Bettenbrock K, Fischer S, Kremling A, Jahreis K, Sauter T, Gilles ED: A quantitative approach to catabolite repression in *Escherichia coli*. J Biol Chem 2006, **281**:2578-2584.

Berrios-Rivera SJ, Bennett GN, San KY: The effect of increasing NADH availability on the redistribution of metabolic fluxes in *Escherichia coli* chemostat cultures. Metab Eng 2002, **4**:230-237.

Chassagnole C, Noisommitt-Rizzi N, Schmid JW, Mauch K, Reuss M: Dynamic modeling of the central carbon metabolism of *Escherichia coli*. Biotechnol Bioeng 2002, **79**:53-73.

Cintolesi A, Clomburg JM, Rigou V, Zygourakis K, Gonzalez R: Quantitative analysis of the fermentative metabolism of glycerol in *Escherichia coli*. Biotechnol Bioeng 2012, **109**:187-198.

Henkel SG, Ter Beek A, Steinsiek S, Stagge S, Bettenbrock K, de Mattos MJT, Sauter T, Sawodny O, Ederer M: Basic regulatory principles of *Escherichia coli*'s electron transport chain for varying oxygen conditions. PLoS ONE 2014, **9**:e107640.

Hoefnagel MHN, Starrenburg MJC, Martens DE, Hugenholtz J, Kleerebezem M, Van Swam II, Bongers R, Westerhoff HV, Snoep JL: Metabolic engineering of lactic acid bacteria, the combined approach: kinetic modelling, metabolic control and experimental analysis. Microbiology 2002, **148**:1003-1013.

Kotte O, Zaugg JB, Heinemann M: Bacterial adaptation through distributed sensing of metabolic fluxes. Mol Syst Biol 2010, **6**:355.

Matsuoka Y, Shimizu K: Catabolite regulation analysis of *Escherichia coli* for acetate overflow mechanism and co-consumption of multiple sugars based on systems biology approach using computer simulation. J Biotechnol 2013, **168**:155-173.

Usuda Y, Nishio Y, Iwatani S, Van Dien SJ, Imaizumi A, Shimbo K, Kageyama N, Iwahata D, Miyano H, Matsui K: Dynamic modeling of *Escherichia coli* metabolic and regulatory systems for amino-acid production. J Biotechnol 2010, **147**:17-30.

Yao R, Hirose Y, Sarkar D, Nakahigashi K, Ye Q, Shimizu K: Catabolic regulation analysis of *Escherichia coli* and its *crp*, *mlc*, *mgsA*, *pgi* and *ptsG* mutants. Microb Cell Fact 2011, **10**:67.
