# Supplementary material for: Modeling and simulation of the redox regulation of the metabolism in Escherichia coli at different oxygen concentrations
Source: Biotechnol Biofuels. 2017 Jul 14;10:183. doi: 10.1186/s13068-017-0867-0 (PMC5512849; doi:10.1186/s13068-017-0867-0)
Supplement: Supplementary file 3 — Additional file 3. Additional results; including additional figures S1–S5. [file 13068_2017_867_MOESM3_ESM.doc]

**Additional file 3**

**
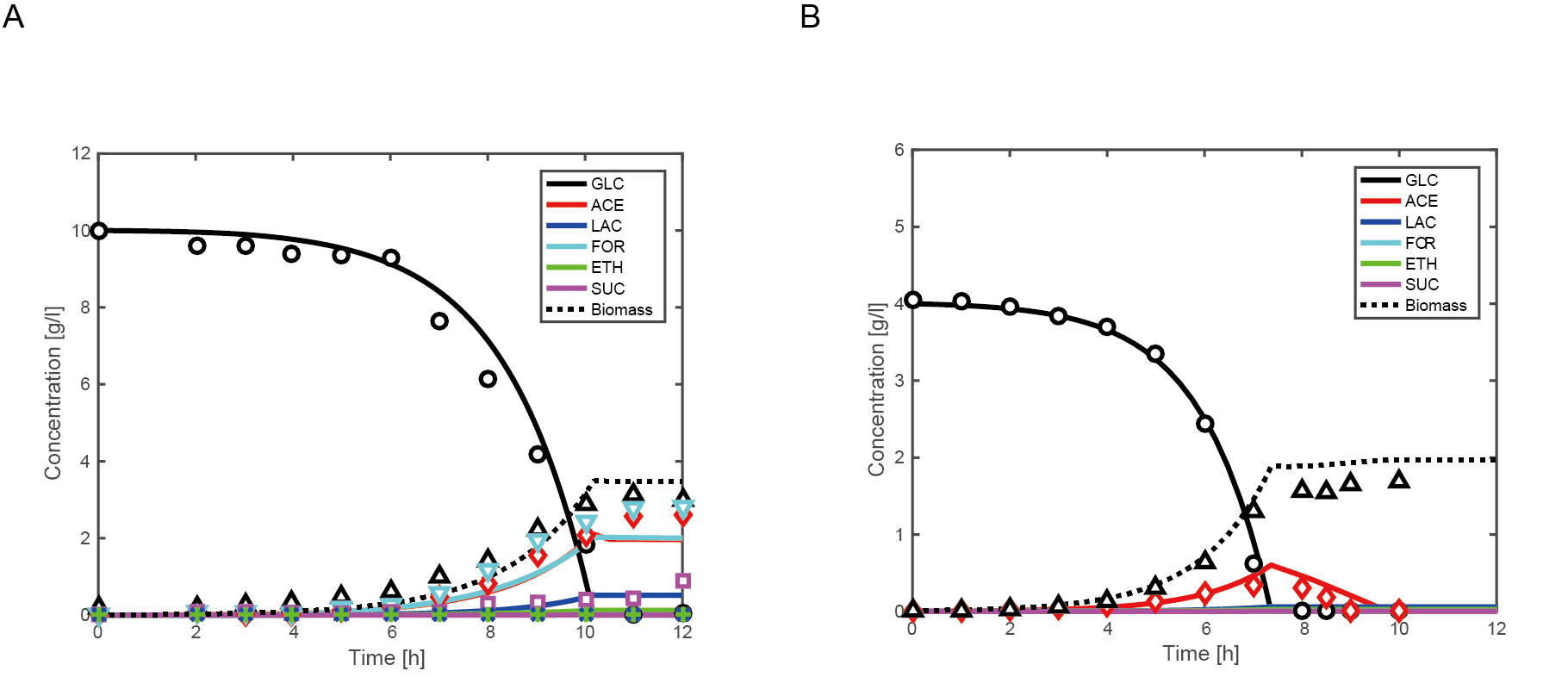
**

**Figure S1. Batch cultivations of wild-type *E. coli* under micro-aerobic (A) and aerobic (B) conditions.** The DO levels were set to 9 and 40% for the simulation under micro-aerobic and aerobic conditions, respectively. Lines show simulation results, and symbols indicate experimental data (Zhu and Shimizu, 2004; Toya et al., 2010): ○, glucose; ◇, acetate; ×, lactate; ▽, formate; ＋, ethanol; □, succinate; △, biomass.

**
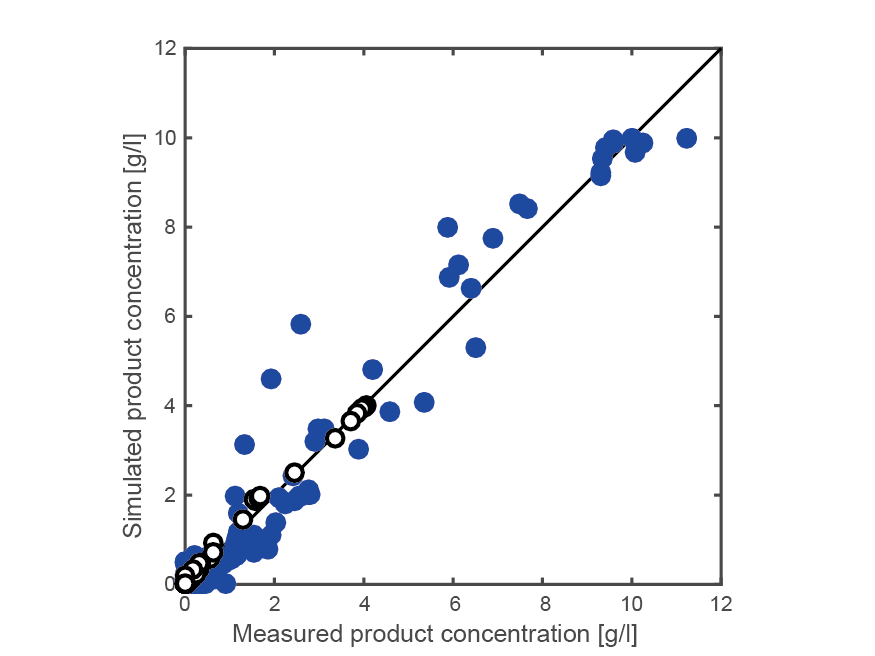
**

**Figure S2. Comparison of the simulated values with the experimental data.** The open and filled symbols represent the data under aerobic conditions (○) and micro-aerobic conditions (●), respectively. The experimental data were taken from (Zhu and Shimizu, 2004; Zhu and Shimizu, 2005; Toya et al., 2012; Toya et al., 2010).


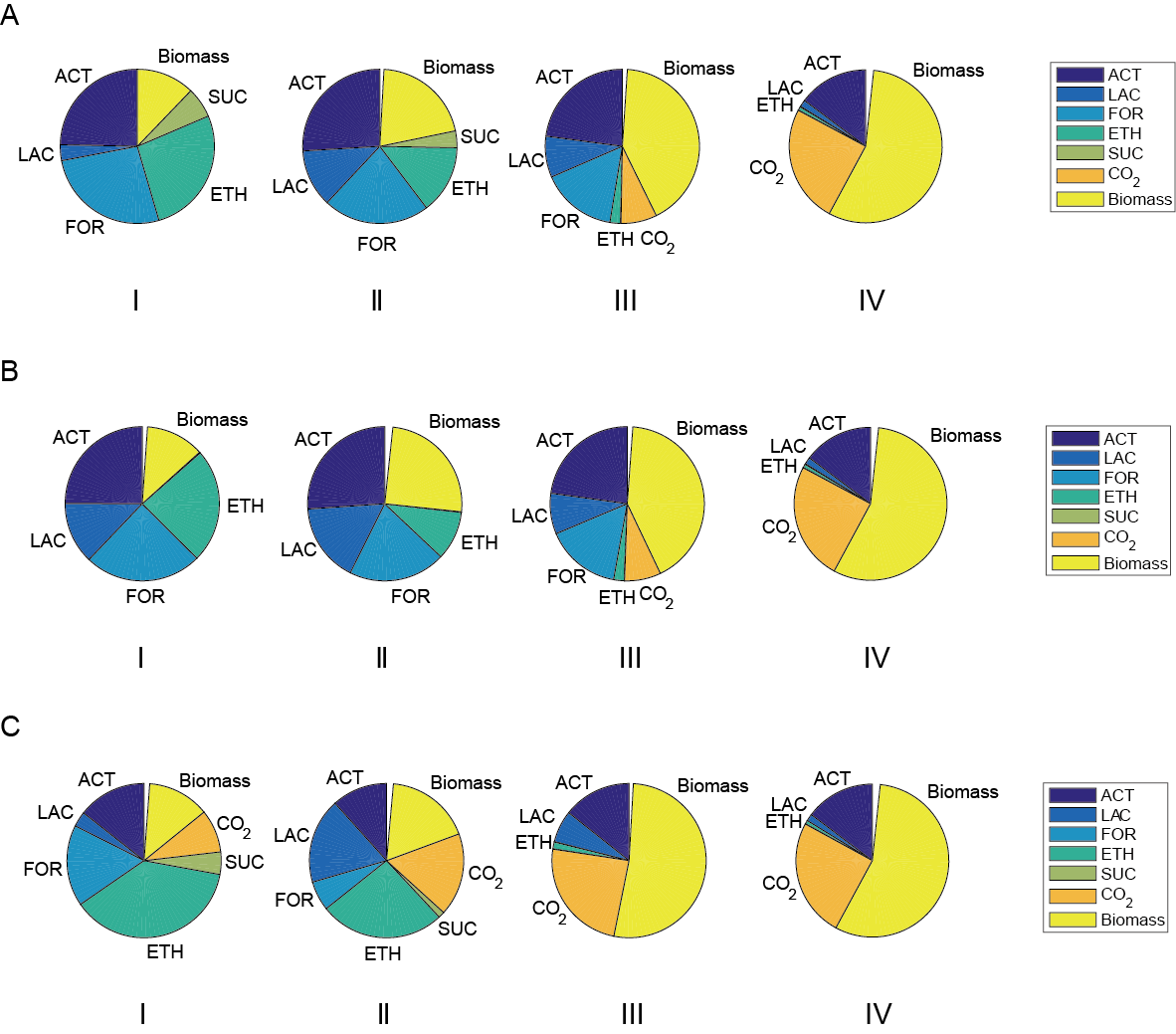


**Figure S3. Carbon balances for fermentative products, CO2, and biomass in the wild-type strain (A), *fnr*-knockout mutant (B), and *arcA*-knockout mutant (C) at different DO levels.** DO levels of 0, 3, 8, and 40% of air saturation were selected as representatives of conditions I, II, III, and IV, respectively.

**A B**

**C D**

**Figure S4. Schematic illustration of the metabolic pathways of wild-type *E. coli* cultivated under conditions I (anaerobic) (A), II (micro-aerobic) (B), III (micro-aerobic) (C), and IV (aerobic) (D).** The important metabolic features are as follows: (D) the NADH/NAD^+^ ratio is low due to the conversion of NADH to NAD^+^ by NADH dehydrogenases at the respiratory chain. Acetate (ACE) is the primary product due to the imbalance between the glycolytic flux and the TCA cycle flux (overflow metabolism); (C) the Cyo flux in the respiratory chain decreases, which results in a decrease in the concentration of quinone (Q), the product of the Cyo reaction and inhibitor of ArcA. The decrease in quinone (as DO level decreases) leads to an increase in the activity of ArcA, which regulates the activities of PDH, Pfl, Nuo, Cyo, Cyd, and most of the TCA cycle enzymes. ArcA activates the Pfl flux to produce formate, while it represses the PDH flux. As ArcA represses the activity of Nuo, the NADH/NAD^+^ ratio increases, which increases NADH-associated reactions involving such enzymes as LDH and ADH to produce lactate (LAC) and ethanol (ETH), respectively; (B) Not only ArcA but also Fnr are activated, both of which regulate the activities of Pfl, Frd, respiratory-chain enzymes (Cyo, Cyd, Nuo, Ndh), and some TCA cycle enzymes. Besides, Fnr regulates the activity of Frd. As Fnr tightly represses the respiratory-chain reactions, the NADH/NAD^+^ ratio increases markedly. The increased NADH/NAD^+^ ratio and Fnr-enhanced Pfl flux work together to promote the ALDH and ADH reactions, producing more ethanol than that under condition (C). The increased NADH/NAD^+^ ratio promotes the reversed reaction of MDH, supplying the substrates to Fnr-activated Frd. This results in enhanced succinate production; and (A) Fnr activity is the highest, and therefore, the respiratory chain reaction stops, and the TCA pathway is branched.


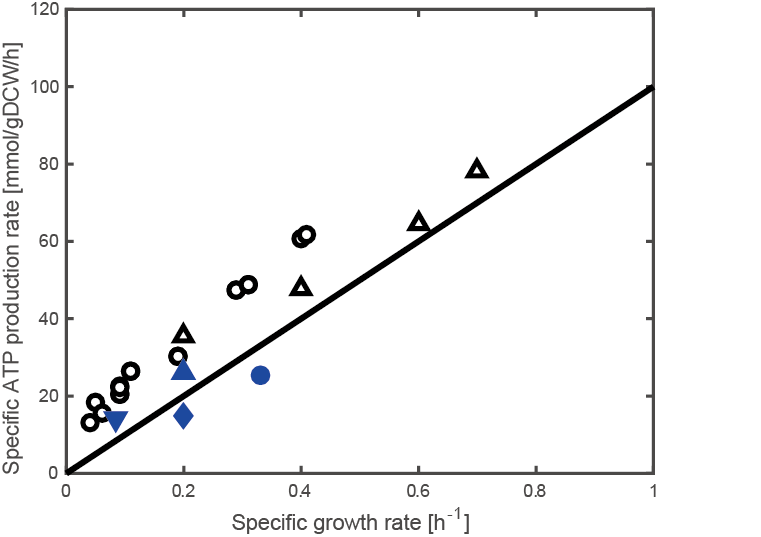


**Figure S5. The relationship between the specific ATP production rate and the specific growth rate.** The solid line represents the estimation using Eq. (3) in the main text. The open and filled symbols are the experimental data of aerobic and micro-aerobic/anaerobic conditions, respectively (○, Nanchen et al. 2006; △, Yao et al. 2011; ●, Gonzalez et al. 2016; ▲, Toya et al. 2012; ◆, Steinsiek et al. 2011; ▼, Zhu and Shimizu 2005).

**References**

Gonzalez JE, Long CP, Antoniewicz MR: Comprehensive analysis of glucose and xylose metabolism in *Escherichia coli* under aerobic and anaerobic conditions by ^13^C metabolic flux analysis. Metab Eng 2016, **39**:9-18.

Nanchen A, Schicker A, Sauer U: Nonlinear dependency of intracellular fluxes on growth rate in miniaturized continuous cultures of *Escherichia coli*. Appl Environ Microbiol 2006, **72**:1164-1172.

Steinsiek S, Frixel S, Stagge S, Bettenbrock K, Sumo: Characterization of *E. coli* MG1655 and *frdA* and *sdhC* mutants at various aerobiosis levels. J Biotechnol 2011, **154**:35-45.

Toya Y, Ishii N, Nakahigashi K, Hirasawa T, Soga T, Tomita M, Shimizu K: ^13^C-metabolic flux analysis for batch culture of *Escherichia coli* and its *pyk* and *pgi* gene knockout mutants based on mass isotopomer distribution of intracellular metabolites. Biotechnol Prog 2010, **26**:975-992.

Toya Y, Nakahigashi K, Tomita M, Shimizu K: Metabolic regulation analysis of wild-type and *arcA* mutant *Escherichia coli* under nitrate conditions using different levels of omics data. Mol Biosyst 2012, **8**:2593-2604.

Yao R, Hirose Y, Sarkar D, Nakahigashi K, Ye Q, Shimizu K: Catabolic regulation analysis of *Escherichia coli* and its *crp*, *mlc*, *mgsA*, *pgi* and *ptsG* mutants. Microb Cell Fact 2011, **10**:67.

Zhu J, Shimizu K: The effect of *pfl* gene knockout on the metabolism for optically pure D-lactate production by *Escherichia coli*. Appl Microbiol Biotechnol 2004, **64**:367-375.

Zhu J, Shimizu K: Effect of a single-gene knockout on the metabolic regulation in *Escherichia coli* for D-lactate production under microaerobic condition. Metab Eng 2005, **7**:104-115.
